# Haematological Safety of Perinatal Zidovudine in Pregnant HIV-1–Infected Women in Thailand: Secondary Analysis of a Randomized Trial

**DOI:** 10.1371/journal.pctr.0020011

**Published:** 2007-04-27

**Authors:** Nelly Briand, Marc Lallemant, Gonzague Jourdain, Somnuek Techapalokul, Preecha Tunthanathip, Surachet Suphanich, Truengta Chanpoo, Patrinee Traisathit, Kenneth McIntosh, Sophie Le Coeur

**Affiliations:** 1 Institut National d'Etudes Démographiques, Paris, France; 2 Institut de Recherche pour le Développement, UMI 174, Program for HIV Prevention and Treatment, Chiang Mai, Thailand; 3 Harvard School of Public Health, Department of Immunology and Infectious Diseases, Boston, Massachusetts, United States of America; 4 Klaeng Hospital, Rayong, Thailand; 5 Bamrasnaradura Hospital, Nonthaburi, Thailand; 6 Somdej Prapinklao Hospital, Bangkok, Thailand; 7 Nopparat Rajathanee Hospital, Bangkok, Thailand; 8 Department of Statistics, Chiang Mai University, Chiang Mai, Thailand; 9 Harvard Medical School, Department of Pediatrics, Boston, Massachusetts, United States of America

## Abstract

**Objectives:**

To respond to the primary safety objective of the Perinatal HIV Prevention Trial 1 (PHPT-1) by studying the evolution of haematological parameters according to zidovudine exposure duration in HIV-1−infected pregnant women.

**Design:**

Multicenter, randomized, double-blind, controlled trial of different durations of zidovudine prophylaxis.

**Setting:**

27 hospitals in Thailand.

**Participants:**

1,436 HIV-infected pregnant women in PHPT-1.

**Intervention:**

Zidovudine prophylaxis initiation at 28 or 35 wk gestation.

**Outcome measures:**

Haemoglobin level, leucocytes, total lymphocyte counts, and absolute neutrophil counts were measured at 26, 32, and 35 wk and at delivery. The evolution of haematological parameters was estimated between 26 and 35 wk (zidovudine/placebo) and between 35 wk and delivery to compare a long versus short zidovudine exposure. For each parameter, linear mixed models were adjusted on baseline sociodemographic variables, HIV clinical stage, CD4 count, and viral load.

**Results:**

Between 26 and 35 wk, haemoglobin, leucocytes, and absolute neutrophil counts decreased in zidovudine-exposed compared to unexposed women (mean difference [95% CI] −0.4 [−0.5 to −0.3], −423 [−703 to −142], −485 [−757 to −213], respectively). However, between 35 wk and delivery, the haematological parameters increased faster in women exposed to long rather than short durations of zidovudine (0.1 [0.0 to 0.1]; 105 [18 to 191]; 147 [59 to 234], respectively). At delivery, the differences were not statistically significant, except for mean haemoglobin level, which remained slightly lower in the long zidovudine treatment group (difference: 0.2 g/dl). Zidovudine had no negative impact on the absolute lymphocyte counts.

**Conclusion:**

Zidovudine initiated at 28 wk gestation rather than 35 wk had a transient negative impact on the evolution of haematological parameters, which was largely reversed by delivery despite continuation of zidovudine. This result provides reassurance about the safety of early initiation of zidovudine prophylaxis during pregnancy to maximize prevention of perinatal HIV.

## Editorial Commentary

**Background:** Pregnant women who are infected with HIV are at high risk of passing on the virus to their unborn baby during pregnancy, labour, and breastfeeding. Around 15%–30% of babies born to HIV-positive women will themselves become infected, if the woman avoids breast-feeding but does not use any other means of preventing the virus from being passed on. However, if a drug, zidovudine (AZT), is given during pregnancy the chance of HIV being passed on to a baby drops from around 23% to around 8%. In some settings it may not be realistic to give the standard course of zidovudine, from 28 weeks of pregnancy, because of its cost and complexity. A number of trials have therefore looked at whether standard-course and short-course zidovudine are equivalent at reducing the risk of passing on HIV from mother to baby. One trial, the Perinatal HIV Prevention Trial-1 (PHPT-1) found that the short treatment course was substantially less effective at preventing HIV from being passed on from mother to baby. Current international guidance therefore recommends starting zidovudine at 28 weeks of pregnancy. However, zidovudine does have several side effects, including anemia; it can also cause a drop in the levels of certain types of white blood cell, and is thought to be toxic to bone marrow. The researchers who had carried out the PHPT-1 trial therefore wanted to do a subsequent analysis of data from that trial to find out whether there were any differences in these safety outcomes between standard and short course zidovudine.

**What the trial shows:** In total 1,436 women were recruited into the trial and assigned to receive either zidovudine from 28 weeks of pregnancy until delivery (standard course; 769 women), or from 35 weeks to delivery (short course; 667). Blood tests were performed at 26, 32, and 35 weeks of pregnancy and then at delivery, and the main outcomes assessed in this secondary analysis were the hemoglobin levels (to check for anemia), and levels of white blood cells, including the levels of two particular types (neutrophils and lymphocytes). The researchers found that standard-course zidovudine resulted in a drop at 35 weeks in the levels of hemoglobin and white blood cells, relative to short-course zidovudine. However, by the time of delivery these levels had recovered and no significant differences could be observed between the two arms of the trial. Women receiving standard-course zidovudine were more likely to experience severe anemia, which although a rare event in both arms of the trial, could have serious outcomes.

**Strengths and limitations:** The original trial from which these data were collected was a relatively large, randomized study and in which there was a low rate of loss to follow up. Although no formal sample size calculation was performed for the analyses presented here, the study probably had sufficient power to detect small differences in the outcomes assessed. A key limitation of this study is that the analyses presented here are a secondary exploration of data from the PHPT-1 trial and should therefore be seen as hypotheses to test in further studies, rather than as definitive conclusions.

**Contribution to the evidence:** The analyses presented here add to the findings of the parent trial, PHPT-1, by providing additional data about the toxicity of zidovudine. Other trials have not clearly established whether there are differences between short- and standard-course zidovudine in terms of their toxicity. The findings support current guidelines recommending standard-course zidovudine therapy for HIV-positive pregnant women. It is also crucial that efforts are made to ensure women worldwide can get access to facilities for monitoring the status of their HIV infection, and then receive highly active antiretroviral therapy when it is needed.

## INTRODUCTION

In 1994, the PACTG 076-ANRS 024 trial showed the dramatic efficacy of zidovudine for the prevention of mother-to-child transmission of HIV: the rate of HIV transmission was reduced from 22.6% to 7.6% [1]. Since the release of this result, prophylactic use of zidovudine has been recommended in all industrialized countries [2]. To reduce the costs and increase the feasibility of this intervention in developing countries, short-course zidovudine, starting at 36 wk of pregnancy, was evaluated [3–7]. However, longer treatment durations proved more efficacious [8] and since 2004, the World Health Organization has recommended zidovudine initiation at 28 wk of gestation or as soon as possible thereafter, in women who do not need treatment for their own health or when antiretroviral combinations are not available [9].

Perinatal zidovudine exposure is associated with transient anaemia and neutropenia [10–13], and bone marrow toxicity of zidovudine, probably related to mitochondrial defects, remains a concern [14–16]. The impact of zidovudine exposure during pregnancy on haematological parameters of HIV-infected women has been assessed in several placebo-controlled studies. While one study showed a lower mean haematocrit at delivery in women exposed to zidovudine [4], other studies failed to demonstrate any effect of zidovudine exposure on haematological parameters [1,5]. However, these studies involved modest sample sizes (*n* = 477 and *n* = 280, respectively), and haematological parameters were compared only at delivery.

## METHODS

### Objective

The objective of the present study was to address the primary safety objective of the Perinatal HIV Prevention Trial 1 (PHPT-1) [8]. The PHPT-1 objective was to compare in HIV-infected women the safety and tolerance of long versus shortened zidovudine regimens for the prevention of mother-to-child transmission. In the present study, we assessed the evolution of haematological parameters, including haemoglobin level, leucocytes, absolute neutrophil counts, and total lymphocyte counts, from 26 wk of gestation to delivery.

### Design

PHPT-1 was a randomized, controlled, double-blind clinical trial comparing the efficacy of various durations of prophylactic zidovudine for the reduction of mother-to-child HIV transmission. Details of this trial (ClinicalTrials.gov [http://clinicaltrials.gov/] identifier: NCT00386230), trial design and results have already been published [8]. The protocol received ethical clearance from the Ethics Committees of the Thai Ministry of Public Health, Chiang Mai University and the Harvard School of Public Health.

### Setting

The study was carried out in 27 hospitals throughout Thailand.

### Participants

The study population was composed of 1,436 HIV-infected pregnant women enrolled in the PHPT-1. To be eligible for PHPT-1, women had to have a haemoglobin level higher than 8 g/dl and an absolute neutrophil count higher than 750/mm^3^.

### Intervention

Pregnant women were randomly assigned to long or short duration of zidovudine. Women assigned to the long group received zidovudine from 28 wk of gestation to delivery. Women assigned to the short group received placebo from 28 wk of gestation and then received zidovudine from 35 wk to delivery.

### Outcome Measures

Haematological measurements were first performed at the pre-entry visit, planned at 26 wk gestation, then at 32 and 35 wk gestation and at delivery upon admission to the maternity unit.

We defined anaemia as haemoglobin level lower than 11 g/dl following the conventional threshold proposed by the WHO guidelines for pregnant women in the third trimester [17]. We also used tables from the National Institutes of Health (NIH), National Institute of Allergy and Infectious Diseases (NIAID), Division of AIDS for grading the severity of anaemia [18]. Mild anaemia (grade 1) was defined as haemoglobin levels between 8.5 and 10.0 g/dl; moderate anaemia (grade 2) between 7.5 and 8.4 g/dl, severe anaemia (grade 3) between 6.5 and 7.4 g/dl and potentially life-threatening anaemia (grade 4) as lower than 6.5 g/dl. We defined leucopenia as leucocyte counts below 4,000/mm^3^; neutropenia as absolute neutrophil count below 1,500/mm^3^; and lymphopenia as absolute lymphocyte count below 1,000/mm^3^.

To identify possible inconsistencies, the haematological values of each woman were plotted in a chart and any value above or below 3 standard deviations of the mean were checked against the original laboratory result slip. Only 5 out of 19,000 measurements were found to be inconsistent and were recorded as missing.

For women who experienced haemorrhage-related transfusion, the haematological parameters immediately before and after transfusion were not taken into account. For women who experienced transfusion unrelated to a haemorrhage, the haematological parameters before transfusion were carried forward until delivery.

### Statistical Methods

The evolution of haematological parameters was examined between 26 wk of gestation and delivery, and the observed means at 26, 32, and 35 wk of gestation and at delivery were compared according to the treatment arms using Student's t-test.

To analyse the evolution of haematological parameters, mixed effects models were used to take into account correlations between measurements within the same woman and variability between women [19]. Because of possible treatment period interaction, instead of considering the treatment period as a whole, haematological evolutions were estimated during two periods: first, between the pre-entry visit (26 wk) and the 35-wk visit; and second, between the 35-wk visit and delivery, while all women were exposed to zidovudine. The regression coefficients were compared using the Wald's test.

The evolution of haematological parameters during the first period were compared between women randomly exposed to zidovudine versus placebo, after adjustment on variables at baseline, including maternal age, body mass index, HIV clinical stage, CD4 count, viral load, parity, education level, type of employment, and region of residence. As the first blood test was scheduled two weeks prior to zidovudine initiation, the zidovudine exposure was used as a time-varying variable.

The evolution of haematological parameters during the second period were compared between the two groups of women according to their previous exposure to zidovudine between 28 to 35 wk of gestation, after adjustment on the variables mentioned above and the value of the considered haematological parameter at 35 wk of gestation.

The predicted values of each haematological parameter were estimated at 35 wk and at delivery. The goodness of fit of the models was checked by plotting the residuals. The *p*-values reported are two-tailed, and an alpha level of 0.05 was used to assess statistical significance.

All analyses were performed using SAS statistical software (version 8.2, SAS Institute, http://www.sas.com).

## RESULTS

### Participants and Baseline Data

**Figure 1:**
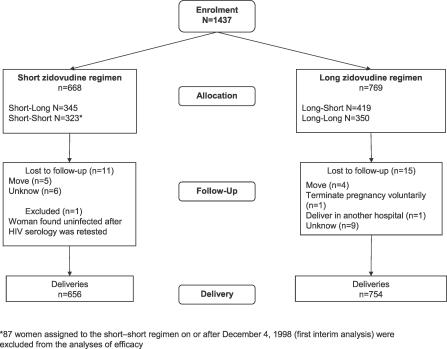
Flow of Participants

The baseline characteristics of 1,436 pregnant HIV-infected women included in the PHPT-1 trial between June 1997 and December 1999 have already been described [8]. The main characteristics are described in Table 1, and participant flow is described in Figure 1. In total, 667 HIV-1 women were randomized to the short zidovudine regimen and 769 women to the long regimen.

**Table 1:**
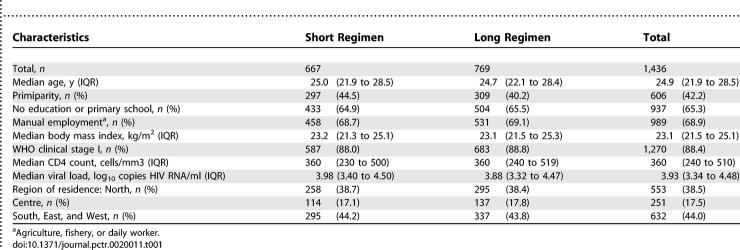
Maternal Characteristics at 26 Wk Gestation According to Randomization Arm

In the entire study population, at pre-entry visit planned at 26 wk of gestation, the median (interquartile range [IQR]) haemoglobin level was 10.7 (10.0 to 11.4) g/dl, the leucocyte count was 8,670 (7,200 to 10,300) cells/mm^3^, the absolute neutrophil count was 6,002 (4,896 to 7,300) cells/mm^3^, and the absolute lymphocyte count was 1,919 (1,476 to 2,425) cells/mm^3^.

Median (IQR) length of gestation was 39.0 (38.0 to 40.0) wk. Median gestational ages at pre-entry and at randomization visit were 25.7 (24.6 to 26.4) wk and 27.9 (27.3 to 28.1) wk, respectively. The median duration of the first period (pre-entry to 35-wk visit) and the second period (35-wk visit to delivery) considered for analysis were 9.0 (8.0 to 9.9) wk and 4.3 (3.9 to 4.8) wk, respectively. The durations of the periods did not differ significantly according to randomization arms.

### Outcomes

**Figure 2:**
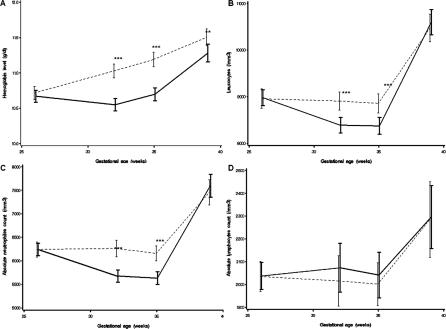
Haematological Parameters between 26 Weeks' Gestation and Delivery in HIV-Infected Women According to Zidovudine Exposure Measurements occurred at the same visit in the two groups. All values are expressed as means and 95% CIs. Comparison of long (zidovudine exposure from 28 wk of gestation) versus short (zidovudine exposure from 35 wk of gestation) regimens was done by Student's t-test. Broken line, short arm; solid line, long arm. *p < 0.05, **p < 0.01, ***p < 0.001. (A) Haemoglobin level. (B) Leucocyte counts. (C) Absolute neutrophil counts. (D) Absolute lymphocyte counts.

The observed evolutions of the mean haemoglobin, leucocyte, absolute neutrophil count, and absolute lymphocyte count, between 26 wk of gestation and delivery, are presented in Figure 2 by randomization arms. The mean haemoglobin level increased steadily between 26 wk and delivery in women randomized to the short regimen (Figure 2A). In women randomized to the long regimen, haemoglobin decreased slightly from 26 to 32 wk, and then increased until delivery. The mean haemoglobin levels, similar at pre-entry in both groups, were significantly lower in women randomized to the long regimen at 32 and 35 wk gestation and, to a lesser extent, at delivery. The mean leucocyte (Figure 2B) and neutrophil (Figure 2C) counts decreased slightly until 35 wk and then increased sharply until delivery, with a more rapid decrease and subsequent increase in women randomized to the long regimen. The mean counts were significantly lower at 32 and 35 wk gestation in women randomized to the long regimen, but did not differ between the two groups at delivery. The lymphocyte counts (Figure 2D) remained stable until 35 wk of gestation and then increased until delivery, with no difference between the two groups.

We have also presented the evolution of the haematological parameters from a safety perspective. Table 2 presents the percentages of women with anaemia at different severity grades (following the WHO guidelines [17] as well as those of the table of the National Institutes of Health, National Institute of Allergy and Infectious Diseases, Division of AIDS [18]) and the percentages of leucopenia, neutropenia, and lymphopenia at pre-entry, at 32 and 35 wk of gestation, and at delivery, according to the randomization arms.

**Table 2:**
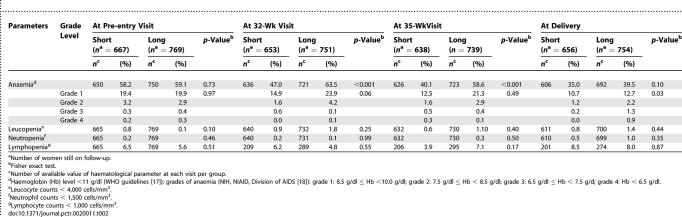
Distribution of Anaemia, Leucopenia, Neutropenia, and Lymphopenia in HIV-Infected Pregnant Women, According to Randomization Arms

Between the pre-entry and 35-wk visits, we found that, after adjustment on variables at baseline, zidovudine intake still had a negative impact on the evolution of haemoglobin level, leucocyte counts, and absolute neutrophil counts, as shown by the mean (95% confidence interval [CI]) decrease associated with exposure to zidovudine, −0.4 (−0.5 to −0.3) g/dl/wk, *p* < 0.001; −423 (−703 to −142) cells/mm^3^/wk, *p* = 0.003; −485 (−757 to −213) cells/mm^3^/wk, *p* < 0.001, respectively (Table 3). Zidovudine exposure, however, had no negative impact on the evolution of absolute lymphocyte counts (166 [0 to 332] cells/mm^3^/wk, *p* = 0.05).

**Table 3:**
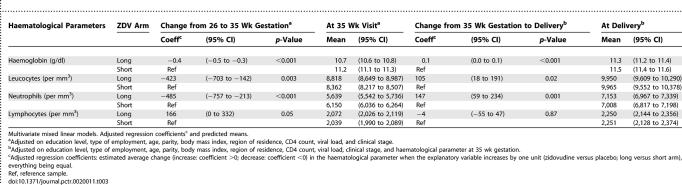
Association between Zidovudine Exposure on the Haematological Measurements Evolution in HIV-Infected Pregnant Women According to Randomization Arms

During the second period, between the 35-wk visit and delivery, after adjustment, the haemoglobin level, leucocyte count, and absolute neutrophil count increased significantly faster in women randomized to the long regimen than women in the short regimen, as shown by the mean (95% CI) increase associated with exposure to zidovudine of 0.1 (0.0 to 0.1) g/dl/wk, *p* < 0.001, 105 (18 to 191) cells/mm^3^/wk, *p* = 0.02, 147 (59 to 234) cells/mm^3^/wk, *p* = 0.001, respectively. There were no differences in the absolute lymphocyte counts (Table 3).

The predicted means are described in Table 3.

Other than zidovudine exposure, the factors independently associated with a negative evolution of haematological parameters were low body mass index, multiparity, low CD4 count, an advanced HIV clinical stage, and high baseline viral load (unpublished data).

### Blood Transfusions

Overall, eight (0.6%) pregnant women received blood transfusions. All were exposed to zidovudine from 28 wk gestation (8/754 versus 0/656, *p* = 0.01). Among these women, two were transfused because of a haemorrhage (at delivery and at 7 d before delivery). One woman with a thalassemia trait received packed red cell transfusions at 45 and 37 d before delivery. The remaining five women were transfused at 0, 2, 4, 25 and 64 d before delivery because of anaemia, including one woman with a haemoglobin level of 4.6 g/dl who delivered a stillborn.

## DISCUSSION

### Interpretation

This study showed that women initiating zidovudine at 28 wk gestation had a transient decrease of haemoglobin level, leucocytes count, and absolute neutrophil count compared to women initiating zidovudine at 35 wk. However, by the time of delivery this initial decrease reversed in women randomized to the long regimen as compared to women randomized in the short regimen, except for haemoglobin levels, which remained lower by 0.2 (0.1 to 0.3) g/dl in women with longer exposure to zidovudine. No negative effect of zidovudine exposure could be detected on the lymphocyte count evolution from 26 wk of gestation to delivery.

This analysis confirms that the severity of haematological toxicity of zidovudine is associated with the duration of exposure. Indeed, at delivery, the percentages of grade 4 anaemia were higher in women randomized to the long regimen as compared with those randomized to the short regimen (0.9% versus 0.0%, respectively). Although adverse events such as severe anaemia, leucopenia, neutropenia, and lymphopenia were uncommon, they should not be taken lightly because of their life-threatening potential. Yet the rate of women who were transfused was of the same magnitude as has been seen in other studies of HIV-uninfected women not receiving zidovudine [20,21]. No woman stopped the study treatment because of haematological toxicity, although one woman exposed to zidovudine from 28 wk of gestation developed severe anaemia and delivered a stillborn. This woman presented at pre-entry biological parameters known to be associated with anaemia, such as low CD4 count (80 cells/mm^3^) and high HIV RNA viral load (5.33 log copies/ml) [22–24].

Women in the study were provided with iron supplementation, according to the national recommendation [25]. However, we had no information about adherence to this recommendation. As the duration of zidovudine exposure was assigned randomly, supplementation is likely to be balanced among the randomized groups in each site and should not have been a source of bias.

More than 90% of the laboratory results for haemoglobin level, leucocyte count, and neutrophil count were available at each visit, with no difference between the randomization arms. Using the complete data method, the sample size remained large enough to demonstrate small statistical differences, even if the clinical significance appears small. For the lymphocyte counts, however, only 33% of the laboratory results were available at 32 and 35 wk of gestation and at delivery. It is possible that the absence of significant difference in lymphocyte evolution between the two groups could be explained by a lack of power.

### Generalizability

The observed evolution pattern of haematological parameters in women randomized to the short regimen was similar to the physiologic evolution generally described in pregnant women during the third trimester of pregnancy [26]. In contrast, in women randomized to the long regimen, the evolution of the haemoglobin, leucocytes, and neutrophils was significantly slowed down until 35 wk, followed by a clear catch-up phenomenon during the last weeks of pregnancy. It should be noted that, in the absence of a control group of women receiving no zidovudine during pregnancy, it is impossible to evaluate the actual impact of zidovudine from 35 wk of gestation until delivery. It is likely that, in the absence of zidovudine exposure, the values of haematological parameters at delivery would have been higher, and this may explain, in part, the apparent convergence of the two groups at delivery.

Interestingly, the 37.4% prevalence of anaemia (haemoglobin <11 g/dl) at delivery, observed in our study was similar to recent national estimates of 38.6% in Thailand [27]. Our results on the potential toxicity of zidovudine during pregnancy cannot be directly compared with other studies because of the different durations of zidovudine exposure, haematological parameters considered, and overall study designs. In a placebo-controlled trial of the efficacy of a short course zidovudine regimen from 36 wk gestation in Thailand, pregnant women exposed to zidovudine had a significantly lower mean haematocrit than unexposed women, 31.9% versus 33.0%, *p* = 0.03 [4]. In our study, the mean haematocrit level at delivery was higher—34.8% in women exposed to zidovudine from 35 wk (unpublished data). The absence of difference in the mean haemoglobin level at delivery in two other placebo-controlled trials may be explained by their relatively limited sample size [1,5].

From 26 wk of gestation to delivery, CD4 counts less than 200 cells/mm^3^, HIV RNA viral load higher than 50,000 copies/ml, and an advanced HIV clinical stage were independently associated with a negative evolution of the haematological parameters. Similar findings have already been reported [22,23], emphasising the need for systematic CD4 testing during pregnancy and access to highly active antiretroviral therapy for immunocompromised women [24]. This result is consistent with the hypothesis that HIV, per se, may be associated with the development of anaemia, presumably because of direct effects of the virus on bone marrow suppression and reduction of erythropoietin production and response [28–32].

### Overall Evidence

The benefit of early zidovudine initiation, namely, its greater efficacy in reducing perinatal transmission [8], is not outweighed by significant disadvantages for the mother in terms of toxicity. As more and more women receive antiretroviral therapies during pregnancy, monitoring of the haematological effects of these therapies remains paramount to ensure their safety.

## SUPPORTING INFORMATION

CONSORT Checklist (27 KB PDF)

Trial Protocol (336 KB PDF)

Text S1 Statistical Analysis Plan (22 KB PDF)

## Abbreviations

CI: confidence interval
IQR: interquartile range
PHPT-1: Perinatal HIV Prevention Trial 1
