# Supplementary material for: Haematological Safety of Perinatal Zidovudine in Pregnant HIV-1–Infected Women in Thailand: Secondary Analysis of a Randomized Trial
Source: PLoS Clin Trials. 2007 Apr 27;2(4):e11. doi: 10.1371/journal.pctr.0020011 (PMC1863515; doi:10.1371/journal.pctr.0020011)
Supplement: CONSORT Checklist — (27 KB PDF) [file pctr.0020011.sd001.pdf]

CONSORT **Checklist of items to include when reporting a randomized trial**

PAPER SECTION And topic	Item	Description	Reported in section
TITLE & ABSTRACT	1	How participants were allocated to interventions (*e.g.*, "random allocation", "randomized", or "randomly assigned").	Methods
INTRODUCTION Background	2	Scientific background and explanation of rationale.	Introduction
METHODS Participants	3	Eligibility criteria for participants and the settings and locations where the data were collected.	Methods
Interventions	4	Precise details of the interventions intended for each group and how and when they were actually administered.	Methods
Objectives	5	Specific objectives and hypotheses.	Methods
Outcomes	6	Clearly defined primary and secondary outcome measures and, when applicable, any methods used to enhance the quality of measurements (*e.g.*, multiple observations, training of assessors).	Methods
Sample size	7	How sample size was determined and, when applicable, explanation of any interim analyses and stopping rules.	NA
Randomization -- Sequence generation	8	Method used to generate the random allocation sequence, including details of any restrictions (*e.g.*, blocking, stratification)	NA
Randomization -- Allocation concealment	9	Method used to implement the random allocation sequence (*e.g.*, numbered containers or central telephone), clarifying whether the sequence was concealed until interventions were assigned.	NA
Randomization -- Implementation	10	Who generated the allocation sequence, who enrolled participants, and who assigned participants to their groups.	NA
Blinding (masking)	11	Whether or not participants, those administering the interventions, and those assessing the outcomes were blinded to group assignment. When relevant, how the success of blinding was evaluated.	Methods
Statistical methods	12	Statistical methods used to compare groups for primary outcome(s); Methods for additional analyses, such as subgroup analyses and adjusted analyses.	Methods
RESULTS Participant flow	13	Flow of participants through each stage (a diagram is strongly recommended). Specifically, for each group report the numbers of participants randomly assigned, receiving intended treatment, completing the study protocol, and analyzed for the primary outcome. Describe protocol deviations from study as planned, together with reasons.	Results
Recruitment	14	Dates defining the periods of recruitment and follow-up.	NA
Baseline data	15	Baseline demographic and clinical characteristics of each group.	Results
Numbers analyzed	16	Number of participants (denominator) in each group included in each analysis and whether the analysis was by "intention-to-treat". State the results in absolute numbers when feasible (*e.g.*, 10/20, not 50%).	Results
Outcomes and estimation	17	For each primary and secondary outcome, a summary of results for each group, and the estimated effect size and its precision (*e.g.*, 95% confidence interval).	Results
Ancillary analyses	18	Address multiplicity by reporting any other analyses performed, including subgroup analyses and adjusted analyses, indicating those pre-specified and those exploratory.	NA
Adverse events	19	All important adverse events or side effects in each intervention	Results

		group.	
DISCUSSION Interpretation	20	Interpretation of the results, taking into account study hypotheses, sources of potential bias or imprecision and the dangers associated with multiplicity of analyses and outcomes.	Discussion
Generalizability	21	Generalizability (external validity) of the trial findings.	Discussion
Overall evidence	22	General interpretation of the results in the context of current evidence.	Discussion
