# Supplementary material for: Haematological Safety of Perinatal Zidovudine in Pregnant HIV-1–Infected Women in Thailand: Secondary Analysis of a Randomized Trial
Source: PLoS Clin Trials. 2007 Apr 27;2(4):e11. doi: 10.1371/journal.pctr.0020011 (PMC1863515; doi:10.1371/journal.pctr.0020011)

Perinatal HIV Prevention Trial
(PHPT-1)

Research Protocol Title:

A SHORT ZIDOVUDINE (ZDV) COURSE
TO PREVENT PERINATAL HIV IN THAILAND

PROTOCOL

Sponsors: NICHD, NIH, R01 - HD 33326
The Ministry of Public Health of the Kingdom of Thailand
Mahidol and Chiang Mai Universities (Bangkok and Chiang Mai, Thailand)
Harvard School of Public Health (Boston, MA, USA)
Institut de Recherche pour le Développement (former ORSTOM), Paris, France

Principal Investigators:

Dr. Vicharn Vithayasai	Dr. Marc Lallemant
Chiang Mai University	Institut de recherche pour le Développement

RESEARCH PLAN

1. SPECIFIC AIMS

The proposed study is a phase II/III double-blind, randomized, controlled equivalence trial comparing the long standard zidovudine (ZDV) regimen with a shortened ZDV regimen for the prevention of mother-to-child HIV-1 transmission.

The study will be carried out in Bangkok and in the northern provinces of Thailand as a collaborative effort between Harvard, Mahidol and Chiang Mai Universities and the Ministry of Public Health of the Kingdom of Thailand.

1.1 Primary Objectives

The study is designed to:

(1) Answer one public health-oriented question related to the optimal duration of ZDV to interrupt perinatal transmission in Thailand and two scientific questions related to the relative importance of maternal and infant duration of therapy.

The three questions to be answered are as follows:

a. Does shortening the duration of both maternal ZDV and infant ZDV treatment increase the risk of perinatal transmission compared with the longer 076-like regimen?

b. Does shortening the duration of maternal ZDV treatment from 3 months prior to delivery to 1 month prior to delivery increase the risk of perinatal transmission?

c. Does shortening the duration of infant ZDV treatment from 6 weeks to 3 to 4 days increase the risk of perinatal transmission?

(2) Assess and compare the safety and tolerance of the long and shortened ZDV regimens.

1.2 Secondary Objectives

(3) To study the pharmacokinetics of oral ZDV administered to women during pregnancy, labor and delivery, as well as their infants.

(4) To study factors associated with mother-to-child HIV transmission (factors related to the mother, the virus, drug exposure, the circumstances of delivery and the newborn).

2. BACKGROUND AND SIGNIFICANCE

2.1 Introduction

Since the beginning of the HIV epidemic, the number of women infected worldwide has steadily increased. Already six and a half million women in subSaharan Africa and 1.5 million women in Southeast Asia are infected with HIV [1]. As yet, there is no efficacious curative treatment for HIV infection and the prognosis for perinatally acquired HIV-1 infection is extremely poor [2-4]. Only prevention of infection in women and interventions aimed at decreasing mother-to-child transmission are therefore capable of having a significant impact on decreasing the incidence of AIDS in children.

Because most mother-to-child transmission of HIV-1 occurs late in pregnancy, and is generally associated with a high virus load and a high level of virus replication, antiretrovirals given for a few weeks before and after birth could dramatically decrease transmission [5].

ACTG 076, a clinical trial conducted in the US and in France to prevent mother-to-child transmission, showed that the administration of zidovudine (ZDV) to the mother during pregnancy and at delivery, and to her infant during the first weeks of life could reduce transmission by as much as two-thirds [6-10]. Moreover, the treatment was well tolerated by both mothers and infants. Because of these results, the trial was interrupted and prophylactic use of ZDV treatment before and after delivery is now considered standard care [11,12]

Even in the US, however, the benefits from this intervention may be diminished since women most at risk of HIV-1 infection traditionally have limited access to medical care, particularly preventive medicine [13]. For that reason, alternative strategies involving other drugs or shorter courses of treatment are still needed [14]. Outreach programs to improve prenatal care as well as access to HIV counseling and testing, are essential so that preventive strategies can benefit women most at risk for HIV. Due to

logistic problems, compliance and cost, the lengthy (18-20 weeks) ACTG 076 regimen may not be optimal in all settings. To improve its use as a public health tool, we need to determine whether the duration of ZDV treatment can be safely reduced in women during pregnancy and/or in their infants without compromising the demonstrated efficacy of the standard 076 ZDV regimen. An efficacious shortened ZDV treatment would improve feasibility, increase safety, increase patient compliance to treatment, reduce treatment cost, decrease side effects, and decrease the risk of ZDV resistance at population and individual levels. Therefore, the chance of its implementation on a large scale in populations that need it the most, both in developed and developing countries, would be greatly enhanced.

2.2 The HIV/AIDS Epidemic in Thailand

Of all Asian countries, Thailand has been the hardest hit by the HIV-1 pandemic. Although the epidemic is still relatively young, the number of HIV-1-infected people in Thailand has or will soon surpass that in the U.S. Almost one million of its 56 million population are thought to be infected [1,15,16].

2.2.1 The Nature of the Epidemic in Thailand

Thailand has two separate epidemics progressing concurrently [16]. The first has developed among intravenous drug users and is caused by HIV-1 subtype B, the virus found in the US and in Europe. The second, of much greater magnitude, is exploding among heterosexual adults and is caused by HIV-1 subtype E, a virus found predominantly in Thailand and transmitted very efficiently through heterosexual intercourse [17-19]. This second epidemic is not confined to high-risk groups such as sex workers and their clients. It is rapidly spreading in the general population [20,21]. Women of childbearing age are becoming just as likely to be infected as men [22]. To date, 90% of HIV-infected pregnant women report heterosexual transmission from spouses or “a steady sexual partner” [20,23].

In Bangkok, the prevalence rate of HIV-1 in pregnant women varies between 1% and 2%. In the northern provinces and around Chiang Mai, the second largest city in the country, HIV-1 prevalence varies from 5% to 10% [24].

In the past few years, AIDS has become a major cause of morbidity and mortality among hospitalized children [25]. AIDS-related mortality among infants and young children has increased nationwide, and may reverse many of the important gains made by effective national child health programs, such as the Control of Diarrheal Diseases and the Expanded Program on Immunization [26].

The six upper districts of the Northern region are the most affected. While these districts represent ~40% of the population of the Northern region, they account for three-fourths of the pregnant women infected with HIV. In 1994, an estimated 4,000 to 6,000 infants were born to HIV-infected mothers in these six districts [24]. The proposed study will be conducted in these districts and a major hospital in Bangkok.

Most perinatally acquired HIV-1 infections in Thailand are caused by subtype E. A transmission rate of 24% (36/151 as of December 1994; 95% CI: 20-27) was reported in a large prospective cohort study in Bangkok (CDC and WHO AIDS Collaborating Center). In this study population, infants were exclusively bottle-fed and diagnosis of perinatally-acquired HIV-infection was based on PCR results when infants were 6 months old with a rate of loss to follow-up of 3% [27].

The following table shows demographic data for the northern region along with results of the latest serosurveillance survey conducted by the Ministry of Public Health among pregnant women [23].

Population data from the 1990 census and serosurveillance data in pregnant women

Region	Total population [1990]	Population in municipality [1990]	Number of live-births [1989]	HIV prevalence [Dec 1993]	HIV prevalence [Apr 1994]	HIV prevalence [Dec 1994]
Upper north provinces of the Northern region						
Chiang Mai	1,376,000	165,000	21,500	7.9%	5.4%	5.1%
Chiang Rai	1,039,000	37,000	14,700	8.0%	6.1%	5.1%
Phayao	504,000	24,000	6,700	10.7%	10.6%	10.7%
Mae Hong Son	173,000	6,000	2,500	2.5%	1.0%	1.1%
Lampang	773,000	44,000	9,600	3.5%	6.3%	3.7%
Lamphun	418,000	15,000	3,300	6.2%	4.6%	3.3%

2.2.2 Government Response

The project we propose will be conducted under the auspices of the Ministry of Public Health and the National AIDS Program. The Royal Thai Government has implemented a multi-sectorial AIDS program since 1987. It involves all government ministries, nongovernmental organizations, multilateral donors and private businesses. Cooperation at the national level among all government ministries is carried out through the National AIDS Prevention and Control Committee chaired by the Prime Minister [28]. The National AIDS Program focuses on 1) Public information, education and prevention: Nationwide campaigns have been conducted using all forms of mass media. Condom use has been promoted and antibody testing is available in all government hospitals and in many private facilities. Since 1989, all transfused blood is screened for HIV antibodies and by donor self-deferral [29]. 2) Human rights and social support: Since 1990, a national counseling plan has been implemented. The government has taken a clear stand against the discrimination of HIV-infected individuals and has promoted voluntary anonymous HIV testing. 3) Promotion of research. Much epidemiological, clinical and socio-behavioral research has already been conducted, the results of which continue to guide interventions and policies. Thailand is committed to actively participate in the global effort to develop and evaluate HIV/AIDS vaccines. 4) Medical treatment and care. In 1994, the government budget for AIDS prevention and care was 46.2 million US$, and will surpass 60 million US$ in 1995 [30]. The use of ZDV was approved in 1987. In 1994, Thailand spent £1.9 million (~3.13 million U.S.$) to purchase ZDV. Since 1991, ZDV has been provided to AIDS patients who cannot afford it through a program by the Ministry of Public Health. The budget devoted by the Ministry of Public Health to purchase antiretrovirals and treatments for opportunistic infections (anti-tuberculosis and fungus drugs) in 1995 will be 8 million US$ [31]. An agreement between the Thai government and the manufacturer of ZDV allows them to purchase the drug in bulk at a competitive price (< $0.50 per 100 mg).

2.3 General Approaches to Interrupting Mother-to-Child Transmission

2.3.1 Factors that Influence Mother-to-Child Transmission

Several factors that may play a role in perinatal transmission have been studied [32].

Disease Stage and Immune Status of Women. Several studies have shown an increased risk of vertical transmission in women who are in advanced stages of HIV disease, or who progress to AIDS during or after pregnancy [33-36]. Seroconversion during pregnancy has also been associated with increased transmission [37]. Furthermore, a relationship between the level of immunodeficiency, as measured by CD4+ cell counts and perinatal transmission has been demonstrated [38-42]. Vitamin A deficiency may also be associated with an increased risk of transmission [43,44].

Maternal Antibodies. Individuals infected with HIV develop neutralizing antibodies. However, these antibodies fail to control infection as mutations in the virus envelope allow the virus to constantly escape the host immune response. Several research teams have associated the presence of antibodies to the principal neutralizing domain of gp120—the V3 region—in pregnant women with a lower risk of transmission to children [45-47]. Other teams failed to reproduce these results [48-53].

Virus Load, Genotype and Phenotype. Several investigators have observed an increased risk of perinatal transmission with high viral load by quantitative PCR or p24 antigenemia [54-60]. Variability among viruses may also play a role. Genetic and phenotypic comparison of the viruses isolated in mothers and their children showed, in some cases, that the viral strains in infants was a distinct subset of those in their mothers [61]. Preliminary data have suggested that non-syncytium inducing (NSI) isolates are preferentially transmitted from mother to infant even when the mother's strain is predominantly syncytium-inducing (SI) [62,63]

Role of the Placenta. HIV infection of placental cells has been observed, but a correlation with subsequent infection of the infant has not been demonstrated. If cell-free viruses are transmitted transplacentally, then placental integrity could play an important protective role in materno-fetal transmission of HIV [65,66]. Cofactors, such as sexually-transmitted diseases, chorioamnionitis, cigarette smoking and drug use could facilitate transplacental transmission of the virus by increasing the placental permeability [35,67].

Mode of Delivery. Increased duration of membrane rupture has being associated with a high risk of transmission [67]. Obstetrical practices such as invasive procedures (e.g. scalp electrodes) could also facilitate virus transmission during the intrapartum period [68]. A meta-analysis of the risk of perinatal transmission, according to the mode of delivery shows a decreased risk with cesarean section compared to vaginal delivery (14% vs. 20%, $p = 0.04$) [69,70].

Newborn Factors. Factors associated with the newborn are less known. They include skin integrity, the role of mucosal exposure to HIV (oral, conjunctival, gastrointestinal), infant immune response and the persistence of maternal cells in newborn circulation [32].

2.3.2 Timing of Transmission

Vertical transmission of HIV can occur in utero, at delivery and during the post-partum period. Possible mechanisms include the passage of free- or cell-associated virus, and/or infection of the placenta.

In Utero. Studies have established that the fetus can be infected as early as eight weeks of gestation [71]. The exact frequency of early transmission is difficult to establish with confidence, however, because risk of false-positive results due to contamination of fetal tissue by maternal blood during the abortion procedure is high, especially when techniques as sensitive as PCR are used to assess infection. The mechanisms involved in transplacental transmission are as yet unclear. In vitro and in vivo data indicate that trophoblast cells are permissive for HIV, but only with a low level of replication, and that late transmission can occur independently of trophoblast-cell infection, via a disrupted placental barrier [72,73]. The absence of perinatal morbidity, the rarity of detectable clinical and immunologic abnormalities at birth, and the difficulties encountered in isolating the virus from newborns who are later found to be infected, however, points to late, peri-partum transmission of HIV.

During Labor and Delivery: A study of twin pregnancies supports the hypothesis of late transmission. Among discordant twins, the first one is infected significantly more often than the second. The first twin may be exposed to HIV infection through proximity to the cervix during the last weeks of pregnancy or have a more traumatic labor than the second twin [74]. This is supported by the fact that elective cesarean section is associated with a reduced risk of perinatal transmission [69,126]. More specific evidence for intrapartum transmission comes from the application of several techniques to diagnose HIV infection early in infancy. HIV isolation, polymerase chain reaction, p24 antigen detection, and HIV-specific IgA and IgM assays on newborn blood have been found negative in a substantial proportion of infants subsequently diagnosed as HIV-infected [75-82]. Using a Markov model to analyze the occurrence over time of positive serology, viral culture and PCR in non breast-fed infants infected perinatally, about 65% of HIV infection in infants was acquired at the very end of pregnancy or at the time of delivery. In the remaining infants who had been infected in utero, it was estimated that 95% had been infected less than two months prior to delivery [83].

Post-partum. Where breastfeeding is prevalent, postnatal transmission of HIV occurs in a significant number of cases. The degree of transmission associated with breastfeeding is difficult to evaluate, though, because infants born to HIV-positive mothers are also exposed to HIV infection during pregnancy and at birth [127]. A meta-analysis of all studies that enrolled mothers who breast-fed and mothers who did not provides an estimate of the additional risk of postnatal transmission attributable to breastfeeding of 14% (CI 95%: 7-22%) [84]. In Thailand, as in industrialized countries, known HIV-infected women are advised not to breast-feed.

2.3.3 Prevention of Mother-to-Child Transmission

Based on data regarding the timing of transmission and factors associated with transmission, several prevention strategies have been envisioned. Antiretrovirals may lower overall maternal virus load, prevent virus replication in the placenta, prevent virus replication in the fetus and/or the infant, or lower the virus load in the maternal genital tract. Passive immune therapy, and/or active immunization may enhance maternal and fetal/infant immune response [6,12,13,85,86]. As a significant number of infants may be infected through mucosal exposure during the birth process itself, disinfection of the birth canal and elective cesarean section have also been suggested as possible ways to reduce transmission [12]. Also, correction of nutrient deficiencies such as low maternal vitamin A may decrease the risk of transmission [44].. Finally, in countries where breastfeeding is the rule, known HIV-infected mothers may be advised not to breast-feed if safe infant formula feeding is feasible [87].

Use of ZDV to Prevent Mother-to-Child Transmission: ACTG 076. To date, only one perinatal intervention has been demonstrated unequivocally to be highly efficacious [6]. The AIDS Clinical Trial Group (ACTG) trial 076 was a Phase III, randomized, double-blind, placebo-controlled clinical trial designed to evaluate the efficacy, safety, and tolerance of ZDV therapy for reducing the risk of transmission of HIV from mother to child. Eligible women were between 14 and 34 weeks of gestation, had baseline CD4 counts greater than 200 cells/mm^3, and had no previous ZDV treatment. They were randomized to receive either ZDV or a placebo. The ZDV regimen consisted of antepartum ZDV (100 mg PO 5 times daily), plus intrapartum IV loading dose of ZDV, for the mother, followed by ZDV (2 mg/kg PO 4 times daily) for six weeks beginning 8 to 12 hours after birth, for the infant. Infants were defined as HIV-infected based on one positive viral culture obtained from peripheral blood. Treatment was well tolerated by both mothers and infants. Reported side effects were balanced between the two randomized groups except for hemoglobin levels, which were slightly lower in infants in the ZDV group. The first interim analysis of the 364 first mother/child pairs showed that the rate of transmission was 8.3% in the group treated with ZDV and 25.5% in the control group (67.5% relative reduction of transmission; p = 0.000006). The trial was immediately interrupted after this analysis to provide all women and infants with the active drug. Further follow-up of mothers and infants is being conducted, to determine if there are any late adverse effects from the treatment (ACTG 288 & 219).

3. A PROPOSED SHORTENED COURSE OF ZDV THERAPY

The goal of ACTG 076 was not to determine the timing of transmission or its mechanism but to test the treatment regimen that was believed to give the best chances of success in preventing perinatal transmission since it covered the pre- and post-natal periods. Most evidence on the timing of perinatal transmission indicates that early transplacental transmission is much less common than previously thought and that most transmission occurs during the last weeks of pregnancy and at delivery. Therefore, a shortened regimen that would include ZDV during the last month of pregnancy, loading dose of ZDV during labor and delivery and oral administration to the infant for one week would likely be as effective as ACTG 076. Such a treatment would cost substantially less and would be considerably more accessible to women. The cost for drug only of the full ACTG regimen was $500 on average for the prenatal treatment, $400 for IV infusion during labor and $12 for treatment of the infant. A prenatal treatment shortened to one month would cost less than $200, oral loading dose as opposed to IV would cost $20 and one week treatment of the infant less than $10. The total cost of the treatment would be less than $230, three to fourfold less than the ACTG 076 regimen. In addition, special agreements between manufacturers and individual countries that purchase ZDV in bulk could cut this cost by at least twofold.

For generalized use of ZDV or other antiretroviral compounds to prevent perinatal transmission, most attention and action has focused on either the U.S. and Europe (ACTG 076), where it is well understood that an efficacious course will be implemented regardless of cost. Conversely, the WHO and certain other international bodies have focused on subSaharan Africa, where use of antiretrovirals will not be possible in the near future because of economic realities. Thailand represents a rare situation and opportunity that truly lies between these two extremes. ZDV is already available there, and it will be used for prevention of perinatal transmission. Nevertheless, there is a clear mandate to simplify the treatment schedule and maximize its efficacy to cost ratio. Although an equivalence trial to test this possibility would not be appropriate for either the U.S. or subSaharan Africa, the results, if equivalency is determined, would have major benefit for both industrialized and developing countries.

The commitment of the Thai government to mobilize significant resources for prevention and treatment of HIV/AIDS is attested by its spending of $3.13 million last year to provide ZDV to HIV-infected patients at an advanced stage of disease. Immediate implementation of a protocol similar to ACTG 076 in the Northern provinces of Thailand, where approximately 6000 infants were born to HIV infected mothers last year, would prevent 1000 cases of pediatric HIV/AIDS and would cost $2.8 million. Two-thirds of this amount would be for the drug and the rest for testing and counseling of all pregnant women. The cost of a shorter regimen would be about $1.3 million.

The cost per case of pediatric HIV infection averted (~$2500) is as cost effective as other interventions already implemented in Thailand. The Japanese encephalitis vaccination (~$5 per dose) is routinely offered to children in the 20 high-risk provinces of the country. Another example is the hepatitis B vaccine. The cost of adding the hepatitis B vaccine to an infant's routine immunization is ~$25 to $50 per DALY (Disability Adjusted Life Year), a unit that takes into account that the health benefit of the intervention will mostly take effect in adulthood (World Bank Report, 1993). While international public health experts have discussed the inclusion of the hepatitis vaccination in the WHO Expanded Program of Immunization since the mid1980s, screening for hepatitis B antigenemia is already part of prenatal care in Thailand, and infants born to HBe antigen-positive mothers are systematically immunized at birth. Using the same unit for comparison, the cost per DALY would be between $50 and $100 for a short ZDV preventive program and between $80 and $130 for an ACTG 076-like intervention . These calculations do not include the hospital and medical cost of pediatric AIDS that would be subtracted from the cost of prevention.

In the United States, the cost of ZDV prophylaxis is estimated to be five times lower than the cost of care [138]. In Thailand, although costs vary widely depending on the type of medical facility where infants are treated, the Ministry of Public Health estimates the yearly cost of an HIV-infected child to be between $500 and $1000. Simply making ZDV available to HIV-infected pregnant women, however, would not assure widespread use and compliance with an ACTG 076-like regimen. HIV testing and counseling programs and professional training to assure safety and compliance would need to be developed and implemented countrywide. In short, even if the Thai government could generate the necessary resources, defining a specific treatment protocol based on ACTG 076 and developing the appropriate infrastructure to implement it presents considerable problems. A shorter, simplified, less costly, and entirely oral regimen of ZDV will likely be more widely accepted and implemented more quickly than an ACTG 076-like regimen in Thailand.

Because of the design of ACTG 076 and the wide variation among the duration of treatment of pregnant women in the trial, ACTG 076 did not answer the question of the most effective duration of maternal treatment. The first analysis of ACTG 076 on 200 treated mothers did not show a relationship between treatment duration and infant outcome. However, the number of infected infants in the treatment arm of ACTG 076 (n=13) may have been too small to demonstrate such a relationship. Even when the final analysis of the trial is undertaken, the number of infected infants is expected to remain low (~20). Even if some association between duration of treatment and effectiveness is identified, its biological significance would be questioned because of possible confounding factors. Women coming late in ACTG 076 who received, on average, a short course of therapy, may also have been more at risk of transmitting the virus to their infant for other reasons. Because of colinearity, observational studies will not be able to sort out the role of such factors.

Currently in the US, HIV-infected women are advised to begin treatment as soon as possible after 14 weeks of pregnancy. Thus, a substantial number of women receive six months of therapy. Although such a long treatment may be unnecessary, or even less effective than a shorter regimen, it is unlikely that additional clinical trials will be undertaken in this country to address this question. Planned and ongoing clinical trials for prevention of perinatal transmission of HIV in the US and Europe are focusing on treatments that can be added to the use of ZDV according to the ACTG 076 protocol. For example, trials are ongoing or in the planning stage to assess whether the use of HIVIG further reduces the rate of transmission in mothers already treated with ZDV, or if delivery by cesarean section further reduces transmission. The addition of a second antiretroviral therapy around delivery is also being considered. Other studies will concentrate on women who come too late to benefit from an ACTG 076 regimen. In populations where AZT is currently not an option, other means to prevent perinatal transmission are tested such as topical virucides, vitamin A supplementation or extremely short courses of antiretrovirals. The study proposed here provides a unique opportunity to answer the question of optimal duration of ZDV treatment to prevent perinatal transmission.

The main question we wish to answer in our study is the following: Can we reduce the duration of prophylactic ZDV treatment without increasing the risk of perinatal transmission of HIV, that is, without compromising the demonstrated efficacy of the standard ACTG 076 ZDV regimen?

To answer these questions, we propose an equivalence trial, where a uniform three-month regimen in the mother and 6 week regimen in the infant will be compared with regimens shortened to one month in the mother and 3-4 days in the infant. To date, there is no evidence to suggest that an ACTG 076-like regimen would be less efficacious in Thailand than in the US in preventing transmission. In Thailand as in the US, the study population will be composed primarily of asymptomatic young women. Based on preliminary data, the rate of HIV perinatal transmission in Bangkok, where women do not breastfeed, is similar to that in the US [6,27]. As in Bangkok, none of the women in this trial will breastfeed their infants. Moreover, one study of response to ZDV in HIV-infected Thai patients suggests that subtype E is as sensitive to ZDV as subtype B [88]. To confirm the ZDV susceptibility of subtypes of HIV-1 circulating in Thailand, fresh isolates from 4 HIV infected Thai adults have been initially tested in our laboratory (Dr. Scott Hammer, New England Deaconess) for phenotypic ZDV sensitivity in the ACTG/Department of Defense (DOD) consensus assay [142,143], a peripheral blood mononuclear cell, p24 antigen based method. These 4 isolates, 2 confirmed as subtype E and 2 as subtype B by PCR amplification of the envelope, had ZDV 50% inhibitory concentrations (IC_{50}s) < 0.01µM, a sensitivity comparable to that reported for fully susceptible isolates. Additional strains are currently being assayed.

Rationale for the Study Design. From the public health perspective, whether the proposed modified treatment regimen (reduced length of treatment in mother and infant) is as effective as a regimen based on ACTG 076 will have a decisive impact on policy decisions. A reduction in treatment for both the mother and the infant would be optimal, since it would significantly reduce the cost and complexity of a perinatal transmission prevention program, and would have a substantial impact on its implementation.

The public health question is therefore best addressed by comparing the transmission rate of the mother-infant pairs who both receive the long treatment ($L_{mother}L_{infant}$) with that for mother-infant pairs who both receive the short treatment ($S_{mother}S_{infant}$). $L_{mother}L_{infant}$ is most similar to the treatment in ACTG 076 and $S_{mother}S_{infant}$ is the full expression of the

proposed modified treatment. This comparison could be made in a simple two-arm clinical trial, but such a trial would not address the scientific question of the separate effects of reducing duration of treatment in mother and the infant.

To achieve the fundamental scientific goal of measuring the separate effects of shortened treatment duration in the mother and shortened treatment duration in the infant, two study designs are possible–a three-arm trial or a 2 x 2 factorial design [140,141]. A three-arm trial with $L_{mother}L_{infant}$, $S_{mother}L_{infant}$, and $S_{mother}S_{infant}$ arms would allow for the following comparisons to be made: $L_{mother}L_{infant}$ to $S_{mother}S_{infant}$, addressing the basic public health question, $L_{mother}L_{infant}$ to $S_{mother}L_{infant}$, which looks at the effect of shortened treatment duration in the mother, holding treatment duration in the infant constant, and $S_{mother}S_{infant}$to $S_{mother}L_{infant}$, which looks at the effect of shortened treatment duration in the infant, holding treatment duration in the mother constant. To achieve acceptable power, the sample size required for the three-arm design would be ~50% larger than for a 2x2 factorial design. We have therefore chosen the factorial design because it addresses the public health and scientific questions with substantially more efficiency than the three-arm design.

The proposed study has a factorial design with four arms:

(1) three months of ZDV treatment in the mothers (the mean duration of ACTG 076) during pregnancy and delivery, and six weeks of ZDV treatment for their infants; L_{mother} L_{infant};
(2) shortened maternal treatment to begin one month before the expected date of delivery with an additional six weeks of ZDV treatment for their infants;S_{mother} L_{infant};
(3) long maternal treatment with treatment of infants shortened to 3 to 4 days; L_{mother} S_{infant}; and
(4) shortened ZDV treatments for both mothers and their infants. S_{mother} S_{infant}.

The design offers two estimates of the main effect of reducing treatment duration in the mother: P_{LL}-P_{SL} and P_{LS}-P_{SS}, the first estimate being the effect of reducing treatment duration in the mother when infants receive six weeks of treatment and the second being the effect of reducing treatment duration in the mother when infants receive 3 to 4 days of treatment. Estimation of the main effect of reducing infant treatment would be completely analogous.

If no interaction is present, these two estimates may be readily combined into a single estimate. If the two estimates of the main effect P_{LL}-P_{SL} and P_{LS}-P_{SS} were different, there would be an interaction between shortened maternal treatment and shortened infant treatment. It is highly unlikely that substantial interaction between maternal and infant treatments will occur since treatment of the infant temporally follows treatment of the mother. From a biological perspective, it is not possible for the infant treatment to have an impact on the effect of reducing treatment length in the mother. Theoretically, varying treatment length in the mother could have an effect on treatment length in the infant if the length of the maternal treatment affected the virus load delivered to the infant or if this treatment induced development of ZDV-resistant viruses. This effect is not likely to be substantial.

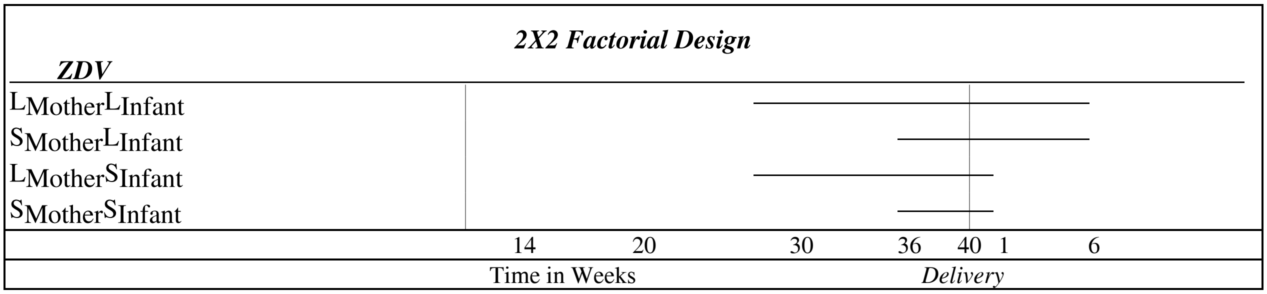

3.2 Rationale for ZDV Treatment Duration and Dosing for Mothers

Pharmacokinetics of ZDV During Pregnancy. Current information on the pharmacokinetics of ZDV in pregnant women is derived from ACTG trial 082, a Phase I pharmacokinetic and safety study of ZDV in the third trimester of pregnancy [89,90]. Asymptomatic HIV-1 infected women, between 28 and 36 weeks of uncomplicated pregnancy, received a single IV dose of 200 mg, followed by an oral dosing regimen of 200 mg every four hours. During labor, ZDV was administered intravenously at a dose of 140 mg every four hours. Following intravenous dosing, the mean ZDV half-life was 1.1 hours, and 1.3 hours after oral dosing. The average bioavailability of ZDV was 60% (range 38% to 93%). The total urinary recovery of ZDV averaged 80% (ZDV and glucuronyl ZDV). ZDV was present in amniotic fluid and vaginal secretions. Data on toxicity and side effects of ZDV in pregnant women was mainly collected in ACTG 076. A full description of these is provided in Appendix 8.1 (original ACTG 076 publication).

Long Treatment Duration for the Mother. A uniform, three-month treatment (beginning at 28 weeks gestation) has been chosen for the long treatment arm. With a standardized, long ZDV treatment, no woman will be treated for more than three months, the risk of toxicity will be significantly reduced and logistics of the trial will be greatly simplified. Finally, because the distribution of treatment in the long and short arms will be uniform, implementation of either the long or short treatment will be much easier than for a protocol that allows variable timing of entry into treatment, as in the case of ACTG 076.

Short Treatment Duration for the Mother. Because most in utero transmission occurs during the last weeks of pregnancy [83], prenatal treatment during the last month is likely to be as efficacious as the long ACTG 076 regimen (3 months on average). Indeed, viral load decreases sharply after inception of treatment. A treatment beginning 4 weeks before the presumed date of delivery would ensure the full effect of ZDV on virus load before delivery even for women delivering pre-term.

Dosage and Mode of Administration. To be practical in the Thai context, the mode of administration of ZDV in the trial will be modified in two ways. Women will receive the same dose of ZDV as in ACTG 076, but administered three times a day (200 mg + 100 mg + 200 mg) instead of five times a day. This mode of administration is becoming widely used. This is justified by the fact that the active phosphorylated form of ZDV has an intracellular half-life two to three times longer than its serum half-life [91,92]. In addition, data from Thailand support the safety and efficacy of such a dosage [88]. More specifically, a randomized clinical trial is currently being conducted in Bangkok to compare the efficacy and tolerance of ZDV at 500 mg a day, administered in two or multiple takes, i.e. 250 mg b.i.d. versus 100 mg t.i.d. + 200 mg hs to ZDV-naive patients, with early ARC and CD4 counts < 400. The analysis of the first 143 patients (73 and 70) showed that both regimens were equally effective and well tolerated. While mean CD4 counts were similar at base line [230 (+/-97) and 232 (+/-99)], the increases were 45% and 44.1% in the two groups, respectively, after two months of treatment. A similar beneficial effect was seen when patients had a very low CD4 count (< 200) or moderately low CD4 count (200-399). Adverse effects, including anemia (3%), nausea and vomiting (8%) and myalgia (2%) resulted in the premature discontinuation of ZDV in 7% of the study population. Compliance was good, with a drop-out rate of 5.6% over 12 months.

The second modification concerns the AZT loading dose during labor, which will be given orally instead of by intravenous infusion in the maternity ward. Based on the pharmacokinetic data above, an oral loading dose of 300 mg with additional doses of 300 mg every 3 hours until delivery would be as effective as the ACTG 076 IV administration (Burroughs Wellcome, Appendix 5.3). Preliminary data show that the pharmacokinetics of oral ZDV dosing during labor do not differ from ZDV during pregnancy [A. Ruff, personal communication and ref. 93]. In addition, oral administration of ZDV during labor substantially reduces treatment cost ($20 instead of $400) and is much more feasible for widespread application in Thailand. Oral administration of ZDV may also allow women to receive the study drug earlier during labor. This reduces the risk that an infant will be born with a low level of ZDV. Women will be told to take a first dose of 300 mg at home, at onset of labor. This will be repeated in the maternity ward every 3 hours until delivery. Since data on the pharmacokinetics of oral administration of AZT at delivery is limited, a phase I pharmacokinetic study will be performed during the pilot phase of the project in the Bangkok site (see pharmacokinetic study section).

3.3 Rationale for ZDV in the Postnatal Period

Pharmacokinetics of ZDV in Newborns and Infants. Current information on the pharmacokinetics of ZDV in infants is derived from ACTG trial 049, a Phase I study to evaluate the safety and pharmacokinetics of intravenous and oral ZDV in infants [94]. Thirty-two asymptomatic infants (less than 90 days old) born to HIV-infected mothers were enrolled. The pharmacokinetics of ZDV were evaluated in each infant after IV or PO administration of ZDV. During the study, doses of ZDV were progressively increased from 2 to 4 mg/kg. The kinetics of ZDV were linear within the dose range of 2 to 4 mg/kg. The total body clearance of ZDV increased with age, from 15.0 ml/min/kg in infants less than 2 weeks old, to 23.4 ml/min kg in older infants. Concurrently, there was a decrease in the half-life after two weeks of age, from 2.5 to 1.6 hours. Oral absorption was satisfactory, and bioavailability varied with age, from 64% in infants aged less than one month, to 86% in those older than one month.

Because of the study design and the limited number of infants infected in the group treated, an analysis of ACTG 076 cannot provide a clear indication as to which components of the ZDV protocol, in utero, intrapartum or postpartum, were most important.

The mode of action of ZDV for preventing maternal transmission is unknown. It may lower the maternal virus load to a level where transmission does not occur. It may also prevent HIV infection from becoming established in the infant. The two mechanisms are not mutually exclusive, i.e. when virus exposure is low, passively-transferred anti-HIV maternal antibodies, in conjunction with ZDV, may abort infection. In fact, the rationale for intrapartum administration of ZDV is not to control viral replication during the last hours of pregnancy, but to ensure that the infant gets an adequate level of ZDV before delivery. In administering ZDV to the infant, the hope is to block the first round of replication should the infant be exposed to the virus at birth, possibly by a mucosal route.

Published data from an uncontrolled study of women treated with ZDV during pregnancy and whose infants were not treated suggests that a shorter course of ZDV in infants or no treatment at all may be as effective as ACTG 076 [95]. Preliminary indications from the ongoing Data Analysis Concept Sheet (DACS) 044 of the Pediatric ACTG, which analyzes data from several cohorts of women in addition to the study from UCLA, also suggest a low transmission rate in women treated with ZDV during pregnancy whose infants were not treated.

On the other hand, several animal models argue for a full ACTG 076 six-week regimen in infants or even longer. These carefully controlled animal studies indicate that the success of post-exposure drug prophylaxis depends on the time interval between virus exposure and the onset of therapy, the size of the inoculum and the presence of a functioning immune system, in particular the CTL immune response [97,98]. In the case of infants, the CTL response to antigens to which they were exposed at birth may not be established until 6-8 weeks after birth [99,100]. Thus, if it is the post-exposure (i.e. the infant portion of the 076 regimen) that is most important, than the full regimen of 6 weeks may be needed.

Experiments with ZDV in HIV cultures have shown that viral DNA decreases with time in quiescent cells and appears to have an in vitro half-life of approximately one day [102]. Approximately one week after infection, the levels and rates of virus rescue following stimulation of quiescent cells are very low. Recent data from C. Loveday et al. show a prompt fall in serum HIV-1 RNA within 1-2 days of treatment in patients treated with ZDV [136]. Given the somewhat contradictory information from these clinical studies, animal experiments and in vitro data, we believe the safest option for a shorter ZDV course in nonbreastfed infants is to give ZDV to infants for 3 to 4 days after delivery. This would allow the drug and perhaps the earliest evidence of immunity to abort infection initiated at the time of delivery. The extracellular half life of ZDV in newborns is 13.0 ± 5.8 hours, while the intracellular half-life is substantially longer [125].

As a standard of care in Thailand, women stay 3 days to one week at the maternity ward depending on the mother's and/or child's health. Therefore, oral administration of ZDV to the infant at the hospital may ensure optimal coverage. Because oral administration of ZDV during labor may not be uniform, this would also ensure appropriate levels of ZDV during this critical period. Direct administration of the drug in the hospital also represents, from the Thai point of view, the most practical treatment scheme. From the study point of view, it also simplifies administration of the randomized treatment, since this will begin at the time the mother at the time the mother and infant are discharged from the hospital.

4. RESEARCH DESIGN AND METHODS

4.1 Study Sites

At least 1556 mother-infant pairs (1400 evaluable mother-infant pairs allowing with a 10% loss to follow up) will be required for the final analysis (see section 4.8). To meet this number, eight sites will be included, seven in the Upper-Northern region of the country, where the prevalence of HIV in pregnant mothers is highest, and one in Bangkok.

All participating centers are large tertiary health care facilities. Each includes a large maternity unit, which attends between 150 and 600 deliveries a month, a nursery, a general pediatric ward and a pediatric intensive care unit in the same hospital complex. In addition, a state of the art pediatric intensive care unit is located within less than 100 km radius (See Map in Appendix 6.3). Every site has a well equipped laboratory where HIV testing (EIA, Western blot), hematology and blood chemistry can be routinely performed. All women and their infants will be taken care of by a single obstetrical and pediatric medical team. The team will consist of obstetricians, pediatricians, counselors, nurses, midwives and social workers. Hospital pharmacists will be in charge of dispensing the treatment drug.

4.2 Study Population

4.2.1 General Characteristics of the Population

The study population will be composed of pregnant women presenting for their first prenatal visit at one of the study sites. The entry point being this first prenatal visit, women will be preselected for access to the study. Since the women will be followed in the clinic they initially chose to attend, we do not anticipate any major difficulty in cohort retention. Generally, maternal and child health is well developed in Thailand [103]. Pre and postnatal care is free in all government hospitals. While 80 to 90% of the population in the northern provinces of Northern Thailand are of Thai origin, several groups have migrated from Burma, China and Laos in the past fifty years. Most of these groups belong to the "Hill tribes" (Karen, Lahu, Hmong, and Lisu among others). Although outreach programs have been implemented, these highland populations still receive limited preventive medical care. They belong to a population that would be difficult to follow. Only a small fraction of them receive prenatal care in the major hospitals where the study will occur. As an entry criterion into the study, all women will be asked whether they will be followed at the same site throughout the duration of the study. More than 50% of the women come for their first prenatal visit during the first four months of pregnancy, except for those at the Bangkok site who tend to come later. The average number of

prenatal visits varies between 5 and 7 across different study sites and practically all women receive complete tetanus immunization (two doses). Syphilis serology and hepatitis B antigenemia are assessed routinely with prevalences ranging from 0.3% to 1% and from 4% to 7%, respectively. The incidence of STDs has dropped sharply since the inception of the AIDS prevention campaigns [104]. Half of the women delivering at the study sites are between 20 and 30 years old. 40% to 70% are primiparous, 20% to 35% come for their second pregnancy and 10% to 16% have had 2 or more pregnancies [Data communicated by coinvestigators].

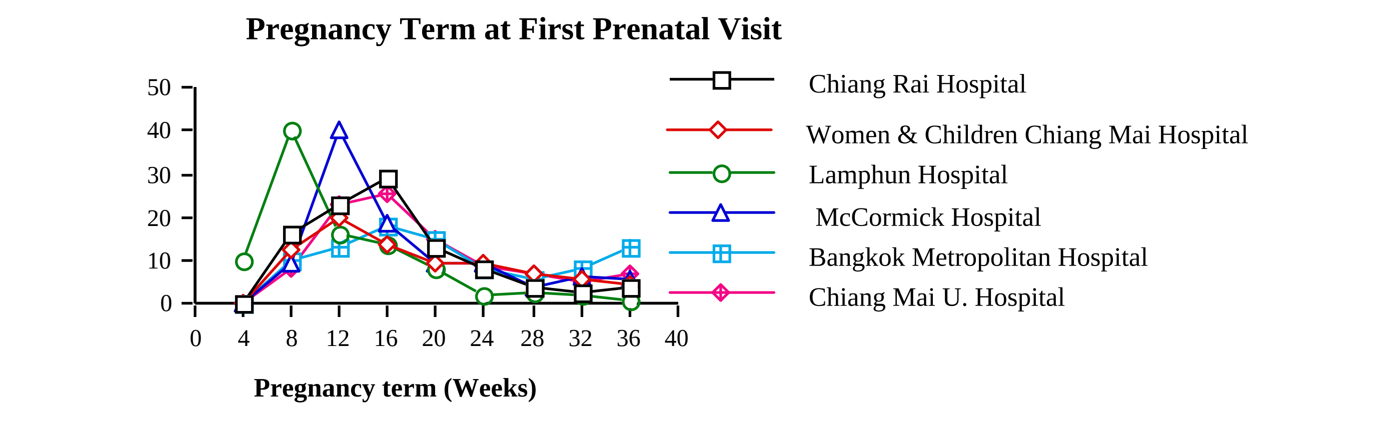

A study by Dr. Panee, the study obstetrician at Chiang Mai University Hospital, compared the health status of rural and urban women. While the average income of urban women was double that of rural women, there were no substantive differences in health status. On average, urban women weighed 51.2 kg and were 150.3 cm in height (Body Mass Index: 22.7) compared with 50.2 kg and 151.7 for rural women (Body Mass Index: 21.8). Anemia (hemoglobin < 10 g) was found in only 5% of women (Average hematocrit: 40.2 and 39.1, respectively and average hemoglobin 14.1 and 13.6, respectively). Average birthweight at each maternity unit varies between 2800-3000 grams reflecting the socioeconomic differences of the population served. 10-13% of the newborns weigh less than 2500 g.

Depending on the mother's and/or child's health, women stay from 3 days to one week at the maternity ward. Immunization in infants is excellent and includes tuberculosis (BCG at birth), diphtheria, tetanus, pretties and polio between two and six months, measles at 9-10 months and Japanese encephalitis (two injections between 12 and 15 months)[103]. In the Bangkok perinatal HIV observational study, the mean age of HIV-positive women was 22 years, 96.1% of them were in the WHO clinical stage 1, 1.8% had a CD4 count less than 200 and 64.0% had a CD4 count between 200 and 500 [27]. HIV-positive pregnant women in the northern provinces are unlikely to differ substantially from HIV-positive pregnant women in Bangkok and we anticipate following mainly asymptomatic women.

4.2.2 Eligibility and Inclusion

Pregnant women presenting at the prenatal clinics will be eligible if they:

- intend to stay in the province after delivery for at least 18 months;
- meet IRB local age of consent and can provide informed consent; and
- have given informed consent to be tested for HIV (Appendix 2.1).

Maternal inclusion criteria consist of:

- evidence of HIV infection (ELISA confirmed by Western blot);
- CD4 count above 200 cells/mm3;
- estimated gestational age of less than 24 weeks based on sonogram results;
- intention to carry pregnancy to term;
- agreement not to breast-feed;
- consent to participate and to be followed for the duration of the study (Appendix 2.2); and
- the following laboratory values within 14 days prior to randomization (about 26 weeks):
  Hemoglobin > 8.0 gm/dl; Absolute neutrophil count > 1000 cells/mm3; Platelets > 100,000 cells/mm3; SGPT< 2.5 x upper limit of normal; Serum creatinine < 1.5 mg/dl (women with a serum creatinine > 1.5 mg/dl must have a measured eight-hour urine creatinine clearance > 70 ml/min).

Maternal exclusion criteria include: a maternal pre-existing condition that contraindicates the use of ZDV; evidence of pre-existing fetal anomalies; a baseline sonogram completed within 14 days prior to randomization that demonstrates during the second trimester an anencephaly, oligohydramnios or during the 3rd trimester an unexplained polyhydramnios, fetal hydrops or

ascites or other evidence of pre-existing in-utero anemia; a history of intolerance to a ZDV dose of < 500 mg/day prior to this pregnancy, resulting in discontinuation of therapy for > 4 weeks; receipt of ZDV during the current pregnancy for any indication, or, women who need ZDV for their own health; and receipt of other antiretroviral agents, passive immunotherapy, anti-HIV vaccines, cytolytic chemotherapeutic agents, radiation therapy, or corticosteroids during this pregnancy (except steroids for less than 7 days).

4.2.3 Recruitment of Study Subjects and Pre-Entry Follow-Up

First Visit: Screening Visit. After a screening questionnaire [Appendix 1.1] and clinical assessment of each woman presenting for prenatal care, those who satisfy eligibility criteria will have the study objectives and design explained to them and they will be asked for their consent for HIV testing. Pre-test counseling will be provided to all women. Confidentiality about the results will be stressed. All women will be informed about the potential benefits and short-term safety of ZDV administered during pregnancy and the neonatal period. They will also be informed that their infant may still become infected and that the long-term risks of ZDV therapy to themselves and their children are unknown (see Appendix 2.1). Blood will be tested by ELISA and confirmed by Western blot. All women will be asked to return in one week for their test result.

Second Visit: Pre-Entry Visit. Women will be informed of their HIV test result and post-test counseling will be provided. All HIV-positive mothers will be asked to participate. Study objectives and design will be explained a second time and their participatory consent will be received [Appendix 2.2]. A detailed background questionnaire will then be administered [Appendix 1.2]. The course of pregnancy will be reviewed for possible exclusion criteria. Each site will maintain records with reasons for subject ineligibility or non-participation. A full clinical examination and a sonogram will be performed. A baseline biological check-up will be performed, including blood counts, hemoglobin electrophoresis, electrolytes, renal and liver function, immune status (CD4 cell counts), blood type, serology from toxoplasmosis and rubella, and an HIV serology to rule out the possibility of misclassification. Lymphocytes and plasma aliquots will be stored for later analysis. The presence of STDs will be assessed. Women will be given an appointment to return to the study clinic one week later.

Third Visit: Women will be given their biological results. Their consent to participate will be confirmed. After receiving in depth information on the treatment schedule, women meeting inclusion criteria will be randomized to one of the four treatment arms. They will receive a month's supply of the study drug, and be given an appointment to return a month later. Investigators will be blinded to the assigned therapy.

4.2.4 Randomization into Groups

Women will be randomized via a computer-generated randomization schedule at the Chiang Mai University site. This site is readily accessible to the other sites by phone, fax and modem 24 hours a day. We will use the statistical package developed for ICAC and ACTG, which performs stratified randomization and dynamic balancing of study sites (See Appendix 5.1). Stratification will be done by baseline CD4 count [> 400 CD4/mm3; < 400 CD4/mm3]. Infants of multiple births will be assigned the same study regimen.

During the antepartum period, mothers will receive 500 mg oral ZDV a day (200 mg +100 mg + 200 mg) until labor. Depending on the treatment arm, mothers will either receive active treatment at 28 weeks or be given a placebo until 4 weeks prior to the estimated date of delivery. At onset of labor, all women will take a 300 mg oral loading dose of ZDV, repeated at 3 hour intervals until delivery (clamp of the umbilical cord). If surgery or anesthesia is needed, women will receive ZDV intravenously according to the ACTG regimen. Infants will receive ZDV syrup four times a day, at a dose of 2 mg/kg as soon as they can tolerate fluids *per os* and if they remain intolerant to fluids for 8-12 hours, they will receive ZDV intravenously (1.5 mg/kg q6h). Depending on the treatment arm, children will receive ZDV either for 6 weeks or 3 to 4 days followed by placebo until 6 weeks of age. Investigators and other associated personnel will be blinded to the assigned therapy.

4.2.5 Post-Randomization Follow-up of Mothers and Children

Maternal visits will be conducted on an outpatient basis for prenatal visits and on an inpatient basis during labor, delivery, and the immediate postpartum period until discharge from the hospital. Infants will be followed initially in the hospital, and later, on an outpatient basis at their participating health center.

Women During Pregnancy. After their pre-entry visit, pregnant women will be followed monthly until the 34th week of pregnancy and then once every 2 weeks until delivery. At each visit [see Appendix 1.3], trained attendants will administer a questionnaire on the women’s health in the preceding month. Adverse events will be recorded. Compliance with treatment will be assessed by pill count. A medical officer will conduct a physical and obstetric examination. A sonogram will be performed at 24 weeks gestation and later as clinically indicated. Every month, hematologic, chemistry, hepatic, and renal toxicity will be checked according to U.S. recommendations [10]. Toxicity may require reducing the dose of ZDV or interrupting the treatment. A non-stress test will be performed at each visit starting at 34 weeks. At each visit, a month's supply of the study drug will be provided to the mother.

Labor and Delivery. At delivery, women will be identified as part of the study by bringing their individual protocol card. Women who did not take the ZDV loading dose at onset of labor as described in the experimental regimen section, will be given the drug immediately after admission. If general anesthesia is anticipated, the loading dose will be administered intravenously [2 mg/kg over one hour followed by a continuous infusion at 1.0 mg/kg/h until delivery]. Circumstances, mode of delivery, and any delivery complications will be recorded [see Appendix 1.4]. It is anticipated that some mothers may deliver at another study site. Coordination with delivery sites will be organized by the investigator at the site where the mothers were enrolled.

Mothers and Newborns after Delivery Shortly after birth, newborns will receive a physical exam by a study pediatrician [see Appendix 1.5]. A blood sample will be taken for PCR (HIV diagnosis) and for assessment of eligibility. The recommendation not to breastfeed will be reiterated. An explanation of formula feeding and infant medication administration, will be given to the mother. Treatment will be given to newborn infants in the nursery as described in the experimental regimen section. If newborns remain intolerant to fluids for 8-12 hours, they will receive ZDV intravenously (1.5 mg/kg q6h). During her stay in the maternity ward, the mother will be instructed by a nurse on how to prepare formula and to administer the study drug. At discharge from the hospital, each woman will be given the study drug appropriate for their infant according to the mother's randomization number, and an appointment will be made for the child's first visit 15 days later. Also at discharge, blood for hematologic and chemical toxicity will be obtained from the infant.

Postnatal Follow-up of Mothers and Children. Every mother and child will be followed once a month for 6 months and once every three months thereafter until 18 months. One final examination will be done at 3 years of age to assess possible late effects of ZDV. This last exam will focus on anthropometry and neural development with the expectation of similar results similar to that for infants in ACTG 076. Preliminary data on 274 uninfected infants (151 ZDV-exposed and 123 placebo) more than 60 months old shows no difference in growth, immune parameters and neurodevelopmental status (E. Connor, personal communication). At each visit, the child's health history since the previous visit will be recorded and compliance to treatment assessed. The child will be examined by a medical officer, blood will be drawn according to the following schedule: PCR and ZDV toxicity at birth, discharge from the hospital, 1 and 3 months, PCR at 6 months based on U.S. recommendations [10]; and HIV serology at 12 and 18 months [See Appendix 1.6]. A supply of powdered milk will be given to the mother at each visit. A medical history, and a clinical and biologic evaluation of the mother will be taken at 3 and 18 months postpartum.

Active Follow-up of Women and Children. We anticipate a low rate of loss to follow-up in this study. Enrolled women will have gone through a process of counseling, HIV testing and explanation of the objectives, potential risks and benefits of the study before actually being enrolled in the study. In addition, they will be followed in the hospital they themselves chose for the follow-up of their pregnancy and postnatal care for themselves and their infant. The DSMB has been asked specifically to review compliance and loss to follow-up and make recommendations as appropriate.

To minimize loss to follow-up, at each monthly visit, women will be asked if they have moved in the preceding month or plan to move. If necessary, and with their informed consent they will be escorted home and the new address will be recorded. In case of a missed scheduled appointment, a social worker will visit the mother's home. In addition, mothers will have access to the study clinic for counseling and care at any time throughout the study period. Children will receive appropriate monitoring and vaccination services at the study site. Mothers will be reimbursed for all transportation and medications costs. Finally, a contact person "in charge of the infant" will be designated by the mother in case she is sick or unable to bring the child to the visit. Since women in the study will have a CD4 count above 200, it is likely that they will remain asymptomatic during the first 6 months post partum.

If a woman or child dies in a health facility, events prior to and at the time of death will be transcribed from hospital records. If death occurs outside the hospital, a standard verbal autopsy form will be administered to assess the cause of death. Infants whose mothers die will continue to be followed during the entire study period. Additional help will be provided to these families through the "Support the Children Foundation" directed by the coPI, Dr. Vicharn Vithayasai. This foundation specializes in helping families to cope with maternal and infant HIV infection and runs several orphanages for children of HIV-infected mothers in northern Thailand. It also has an outreach program in the villages to assist women in dealing with maternal illness and infected infants (See Appendix 6.2).

Longer Term Follow-up of Infants. While ZDV does not appear to cause birth defects [105,106], children will be seen for a final visit at 3 years of age to assess possible late effects. ACTG 219 specifically looks at long term effects of pre and postnatal ZDV exposure in children looking in particular at survival, growth, nutritional status, neurologic and neuropsychologic disorders, organ-system toxicities and cancers. This trial may provide information that would be important to confirm in the Thai cohort of infants. Preliminary results from this study are reassuring.

Concurrent Medications and Treatments: PCP prophylaxis. Trimetroprime/Sulfamethoxazole 5 mg /kg once a day three times a week will be offered to all infants from one month of age when the risk of PCP increases drastically [107,108,139] until 4

months when an assessment of infectious status can be made with reasonable confidence [109]. At this age, infants with two negative PCR tests (at 1 and 3 months), clinically well and with normal CD4 counts will be discontinued from the drug.

Other Prescriptions. All prescription and non-prescription medications received by the mother during pregnancy, and medications received by the infants during the first six weeks of the study will be recorded. Mothers and infants may receive all medications/treatments as required for normal obstetrical management of the HIV-infected woman except for other antiretroviral, passive immunotherapy, or anti-HIV vaccine agents. Drugs that are metabolized by hepatic glucuronidation may alter the metabolism of ZDV and will be used with caution. All infants will receive standard well-child care throughout the study by their primary physician.

Immunizations. Children will be immunized according to current recommendations of Thailand's Ministry of Public Health.

Clinical and Biological Follow-Up of Mothers and Infants

MOTHERS*	1st vst	2st vst	3rd vst		28 wks	32 wks	36 wks	38 wks	Deli-very	1/2 mo	1 mo	2 mo	3 mo	4 mo	6 mo	9 mo	12 mo	15 mo	18 mo
Questionnaire	X	X			X	X	X	X	X				X						X
Physical Exam	X	X			X	X	X	X	X				X						X
Obstetric Exam	X				X	X	X	X	X										
Sonogram		X		**R**															
Non stress test				**A**		X	X	X											
Counseling	X	X	X	**N**					X				X						
Compliance assmt.†				**D**	X	X	X	X	X	X	X	X							
HIV Serology	X	X		**O**															
Hematology§		X		**M**	X		X		X				X						
Chemistry‡		X		**I**	X		X		X				X						
CD4 & CD8 subsets		X		**Z**									X						X
ICD-p24 antigenemia		X		**A**					X				X						
Stored cells (virology)		X		**T**					X				X						
Stored plasma				**I**					X				X						
Hb Electrophoresis		X		**O**															
STD screening//		X		**N**															
HBV/Tetanus vaccine						X	X												
FORMS	Scree ning	Pre-Entry	Entry		Pre natal	Pre natal	Pre natal	Pre natal	Deli very				Post natal						Post natal

* First visit may occur anytime during pregnancy until the 28th week. Randomization may occur anytime between 24 and 28 weeks. Women will attend these antenatal visits depending upon the date of their randomization.

† Compliance for study drug will be assessed by pill count at every visit and by MCV; random testing for serum ZDV levels will be performed; Compliance for bottle feeding will be assessed by asking the mother and breast examination.

§ Hematology includes: Hemoglobin (g/dl), Hematocrit (%), RBC (million/ul), MCV (microns) WBC (10^3/cu mm), Platelets (10^3/cu mm), Reticulocyte count (%).

‡ Chemistry includes: Na, K, Cl, Ca, Mg, Ph, CO2, Alk Phosp, SGOT, SGPT, LDH, Urea Nitrogen, Creatinine, Uric Acid, Glucose, Triglycerides, Total Bilirubin, Total protein, Albumin, Globulin, Amylase, CPK.

// STD screening: Serodiagnosis for syphilis & chlamydia, culture for gonoccoci, smears and gram staining.

INFANTS	Delivery	1/2 mo	1 mo	2 mo	3 mo	4 mo	6 mo	9 mo	12 mo	15 mo	18 mo	36 mo
Physical Examination	X	X	X	X	X	X	X	X	X	X	X	X
Hematology	D 0/4		X		X							
Chemistry	D 0/4		X									
PCR (HIV diagnosis)	X		X		X		X					
HIV serology								X	X	X	X	
CD4 & CD8 subsets					X							
Stored serum	X				X		X	X	X	X	X	X*
Stored cells	X				X		X	X	X	X	X	X*
BCG Immunization	X											
Hepatitis B immunization	X		X				X					
DPT/IPV Immunization				X	X	X					X	
MMR Immunization							X				X	
Japanese Encephalitis											X	
FORMS	Newborn	Infant 1/2	Infant 1	Infant 2	Infant 3	Infant 4	Infant 6	Infant 9	Infant 12	Infant 15	Infant 18	Infant 36

* In infected infants only.

4.3 Laboratory Investigations

All laboratory tests including ELISA, Western blot, T4 cell subsets, hematology, chemistries, and bacteriology will be performed in the laboratories of each study site, according to schedule. The WHO Collaborating Center on AIDS (Dr. Chantapong Wasi; Departments of Microbiology and Immunology of Mahidol University at Siriraj Hospital in Bangkok, and of Chiang Mai University Hospital) will ensure training and quality control. Technicians from all sites will be trained to use the same standardized techniques and reagents kits. Bangkok and Chiang Mai sites will ensure quality control by randomly testing sample aliquots regularly sent to them and by providing panels of samples to be tested.

All procedures including questionnaires, clinical follow-up, toxicity monitoring and laboratory evaluations are provided in the appendices and are listed in the following tables:

4.4. Compliance Assessment
Compliance with treatment will be assessed by three means:
1. Pill count (syrup consumption) at each patient visit.
2. Mean corpuscular volume (data recorded blindly by a laboratory technician and provided to the DSMB).
3. Determination of serum ZDV levels (performed blindly on randomly chosen serum samples and results provided directly to the DSMB). To interpret serum levels, the time since last dosing will be recorded at the time of blood drawing (See pharmacockinetic/dynamic substudy).

Compliance with Bottle Feeding. Although Thailand supports breastfeeding through the "UNICEF Baby Friendly Hospital Initiative," known HIV-positive mothers are advised not to breastfeed. To help HIV-infected women comply with this recommendation, the Ministry of Public Health has initiated a program to distribute powdered milk. This program was initiated in 1993 and funding was doubled this year.

Acceptance of bottle feeding is one inclusion criteria for the study. The issue of breastfeeding will be discussed with women at enrollment and those who agree to participate will be further counseled about the risk of transmission through breastfeeding. Formula will be provided to them and their nursing practices will be assessed at each postnatal maternal or infant follow-up visit. Breast examination will be part of the post-partum follow-up. Data on compliance to breastfeeding will be provided to the DSMB and will be taken into account in a secondary analysis of the data. Intention to treat, however is the primary strategy for the analysis of this trial. As a standard of care in Thailand, mothers stay in the hospital from 3 day s to 1 week. This greatly facilitates teaching of bottle-feeding and study drug administration.

Drug Supply and Storage. The study drugs for this trial will be supplied by Burroughs-Wellcome and will be distributed to the study sites pharmacists through the central pharmacies in Bangkok and Chiang Mai. Every site pharmacist will maintain complete records of all study drugs received. They will ensure that it is properly pre-packaged and pre-labeled. During outpatient therapy the pharmacists will dispense blinded study drugs to patients. All study drugs not dispensed to mothers will be returned by pharmacists to the Chiang Mai or Bangkok dispatch centers. Site implementation plans are currently being formalized.

4.5 Assessment of Safety and Tolerance of ZDV Therapy
Safety and tolerance of ZDV therapy in both treatment regimen groups will be evaluated by examining adverse experiences, and by laboratory and clinical safety data in mothers and infants (See Appendix 4).

Adverse Experiences and Treatment Discontinuation. Adverse experiences will be reported as either associated with ZDV use or not based on the investigator's judgment. Any serious adverse event [see Appendix 4.2] that occurs during this study will be reported to the study monitoring board within 24 hours, whether or not this reaction is considered to be related to the investigation drug. Mothers or infants who experience a serious adverse event will be evaluated within 24 hours of discontinuation of the study drug. All abnormalities observed in the infant at time of birth and during follow-up including abnormal laboratory evaluations, developmental abnormalities and congenital anomalies will be reported. Chiang Mai University Hospital, the coordinating site can be reached 24 hours a day by peripheral sites either by phone, fax, E-mail and through a paging system. Dr. Prakong in the Department of Internal Medicine in Chiang Mai will be the primary contact person. For obstetrical emergencies, Dr. Panee (CM University Department of Obstetrics) or her designee will be the contact person, and for pediatric emergencies, Dr. Prapasri (C.M. University Department of Pediatrics, ICU) or her designee will be the contact. The scheme for reporting adverse events is provided in Appendix 10.

Management of toxicities and dose modifications for mothers and children will be managed according to the ACTG 076 guidelines (See Appendix 4.1 and 4.2). These guidelines will be reviewed with the clinical staff by Dr. K. MacIntosh and R. Tuomala during the on-site training that will be organized prior to starting the study.

4.6. Definition of Main Endpoints

Definitive Infection Status in Infants. Children will be considered HIV-positive if 2 samples are positive by PCR analysis. This early determination of HIV status in infants will allow for an early estimation of the rate of transmission in the study groups and for interruption of the study analysis if the interim analysis shows a statistically significant inferiority of one or more of the treatment regimens. HIV status will be confirmed by IgG serology at 18 months (see Appendix 5.2).

Complementary analysis will use other working definitions of HIV infection in infants, in particular the Ghent consensus definition for intervention studies in developing countries [110].

Timing of Transmission: Transplacental. Infants will be defined as infected in utero if positive PCR culture is found in a peripheral blood sample drawn within the first 48 hours of life [111].

Transmission at Delivery. All infants with a negative PCR at birth but who become positive later will be defined as infected at around the time of birth [111].

Safety of ZDV Treatment for Mother and Child. Clinical and biologic toxicity will be evaluated according to standard assessment criteria (see Appendix 4.1)

Assessment of Cofactors. Although randomization will be the first step to balance known and unknown covariates between treatment groups, we will compare the distribution of these factors among treatment groups. Should an imbalance be detected, the analysis would stratify on these factors. Useful information about selected covariates will be gained by performing Cox and logistic regression analyses as secondary analyses of the study. Confounding factors such as STDs, clinical stage of disease, CD4 count, duration of ruptured membranes, mode of delivery, duration of labor, newborn skin integrity, cigarette smoking, illicit drug use (which we assume will be extremely rare), chorioamnionitis and gestational age will be evaluated and taken into account in the analysis.

4.7 Data Management

Prior to the opening of the study, the manual of operation will be reviewed by all research team members. One training session will focus on the impact of poor data collection and non-compliance to the protocol. A bi-monthly newsletter/report will be used to highlight and further clarify those issues. The forms used to record clinical and biological data are provided in Appendices 1.1 to 1.6. Every form will pass through the following steps: administration, checking (on site before submission to the central data management center), complete entry, verification, and filing (in Chiang Mai). All forms will be reviewed for completeness, discrepant responses, inappropriate responses, and illogical responses. Forms will be checked by the study site team as soon as possible after they are filled out. Either the obstetrician or pediatrician in charge at the study site will be responsible for checking the forms before copies are sent in batches to the central keying location. They will also be responsible for answering queries. Forms passing the review will be sent in batches for data entry at Chiang Mai University. The data entry and management program to be used on site will be written by N. McGrath, a senior statistician who has been in charge of the data management for several years at SDAC. The program will check for duplicates and examine the data set for range checks on each field within a form, consistency checks between fields on the same form, and consistency checks between fields on different forms. The program will also list forms that are inappropriately missing. In case of incoherent or missing data, the study sites team will be immediately contacted and, if necessary the form returned to them for correction. Site visits will be made to monitor the quality of data collected in the research records, the accuracy of the data entered into the database, and to determine that all regulatory requirements surrounding clinical trials are met. Key data items will be verified on a randomly selected sample of forms by confirming data from the original medical and laboratory records. An audit trail will be maintained for all changes made to the original data set.

4.8 Statistical Methods

The primary objective of this study is to determine whether there is a difference in HIV perinatal transmission rates resulting from different durations of ZDV treatment to mothers or their infants. Specifically, we are interested in the following hypotheses:

1. Does shortening the duration of both maternal ZDV and infant ZDV increase the risk of perinatal transmission compared with the longer 076-like regimen?
2. Does shortening the duration of maternal ZDV from 3 months prior to delivery to 1 month prior to delivery increase the risk of perinatal transmission?
3. Does shortening the duration of infant ZDV from 6 weeks to 1 week increase the risk of perinatal transmission?

The factorial design of this study allows direct comparisons of the treatment arms (hypothesis 1), as well as an assessment of the relative efficacies of earlier versus later initiation of ZDV in mothers, and shorter versus longer duration of ZDV therapy in

infants (hypotheses 2 and 3). Furthermore, dependency of the treatment regimens in mothers and children can be assessed. However, as discussed before, additive interaction between treatments – i.e. the relative efficacy of treatment in the infant depending on onset of treatment in the mother – is highly unlikely.

Power and Sample Size. Patients will be randomized to receive one of four treatment regimens. The diagram below shows the four treatment arms in a 2 x 2 factorial design with the projected number of evaluable patients per treatment group.

Maternal Treatment Duration	Infant Treatment Duration	
	Long	Short
Long	$L_{mother}L_{infant}$ n=466	$L_{mother}S_{infant}$ n=233
Short	$S_{mother}L_{infant}$ n=233	$S_{mother}S_{infant}$ n=466

The first question will be answered by comparing the transmission probability for the $L_{mother}L_{infant}$ arm to that of the $S_{mother}S_{infant}$ arm in pairwise fashion. The design (sample size and unbalanced randomization) gives special attention to this pairwise comparison. The study also uses a 2 x 2 factorial design to address the two scientific questions, simultaneously taking advantage of all randomized patients.

The Public Health Question. The public health oriented question is of critical importance and will be given special attention in the design and analysis. In particular, the primary analysis for sample size determination will be the pairwise comparison between the 076-like ZDV regimen and the regimen with both maternal and infant treatment duration shortened. To strengthen the statistical power of this pairwise comparison, we will unbalance the randomization so that two-thirds of the patients receive either $L_{mother}L_{infant}$ or $S_{mother}S_{infant}$, and one-third of the patients receive either $S_{mother}L_{infant}$ or $L_{mother}S_{infant}$.

The answer to the first question has public health implications with respect to adopting a composite regimen with both maternal and infant treatments shortened. The study is designed as an "equivalence trial". The proposed study is designed to test for equivalence between treatments, i.e. that a shorter treatment duration in either the mother or the infant is <u>not worse</u> than the standard long treatment by more than a predefined difference, d, that is both relevant scientifically/clinically and from the public health perspective of Thailand. The short and long regimens will be considered equivalent for practical purposes if the difference between transmission rates is smaller than δ [113,114].

Determination of the critical difference in transmission rate between treatment arms, δ. There are two different perspectives that influenced our choice of study design. From the public health perspective, the primary issue is the reduction of treatment length to diminish cost and increase feasibility of widespread implementation. From the scientific perspective, the key question is whether shortening the duration of ZDV treatment in either the mother or the infant increases the risk of perinatal transmission of HIV, and if so, which reduction, in the mother or the infant, is responsible for the increased risk.

Where resources for a specific objective are limited, policy options are usually determined by calculating the cost per case of HIV infection averted with a cheaper program, and comparing this with the cost per case averted with a more expensive, albeit more effective program [138]. Using a cost-effectiveness analysis, we have calculated that in Thailand, based on a realistic presumption of 3% HIV prevalence among pregnant women in the general population, a 1 month prepartum regimen of ZDV that has a transmission rate no more than 6% higher than a 3 month regimen is clearly more cost effective. The savings gained from using the shorter treatment may be used to extend the program to more women. On the other hand, if the difference in transmission rate between shorter and longer regimens is greater than 6%, the longer, more expensive regimen would be preferred. Therefore, we want to detect a minimum difference, δ = 6%, in transmission rates between women/infant pairs randomized to the standard long treatment, L_{mother} L_{infant}, and those randomized to the shortest treatment regimen, S_{mother} S_{infant}.

Cost effectiveness of long vs. short mother/infant ZDV.

- The average prevalence rate in the target population is 3%;
- Cost of L_{mother} L_{infant} regimen in Thailand (including 15% distribution) is \$306 and that of S_{mother} S_{infant} is \$114;
- The cost of testing and counseling of the target population is \$5;
- The transmission rate without treatment is 25% and the rate with L_{mother} L_{infant} treatment is 8%.

Based on these assumptions, the cost per case averted is \$2780 for the $L_{mother}L_{infant}$ treatment arm. If the transmission rate with the shorter treatment is higher by 1%, then the cost per case averted with this treatment is \$1757, significantly less than the

long treatment. As shown in the table below, when the difference in efficacy between the treatments increases, the cost effectiveness of the short treatment decreases up to a point where the longer treatment becomes more cost-effective.

Treatment	Transmission Rate	Lives saved per 1000	Cost per life saved	Delta	Incremental cost
None	.25	-	-	-	-
Lmother-Linfant	.08	170	**2780**	.-	-
Smother-Sinfant	.09	160	1757	.01	19147
Smother-Sinfant	.10	150	1874	.02	9573
Smother-Sinfant	.11	140	2008	.03	6382
Smother-Sinfant	.12	130	2162	.04	4786
Smother-Sinfant	.13	120	2343	.05	3829
Smother-Sinfant	.14	110	**2555**	**.06**	**3191**
Smother-Sinfant	.15	100	2811	.07	2735

Testing for Equivalence: To test for equivalence, the null hypothesis is that the standard treatment is better than the new by the predetermined δ or more. The alternative hypothesis is that the difference between the effect of the 2 treatments is less than δ, the new treatment is at least equivalent to the long treatment [131].

The first type of error that we are concerned about is to declare equivalence of treatments when the true difference is greater than δ. Therefore, the sample size will be calculated so that this risk, α, is no more than 5%. Another potential class of error would be to conclude that the two treatments are unequivalent when the true difference is less than δ. This would result in choosing to keep the standard treatment when the new treatment is as good. In this trial, the sample size will be such that the risk of missed equivalence is less than 10%.

H_0: $\pi_l \leq \pi_s - \delta$, Hypothesis of a specified difference H_1: $\pi_l > \pi_s - \delta$, Hypothesis of equivalence

Sample size in each of two groups: $$N = \frac{(z_{1-\alpha} + z_{1-\beta})^2 \, (\pi l(1-\pi l) + \pi s(1-\pi s))}{(\pi l - \pi s + \delta)^2}$$

π_l and π_s are the true transmission rates for long and short treatment (set equal under H_1)

$z_{1-\alpha}$ and $z_{1-\beta}$ are the upper percentage points of the standard normal distribution

Sample Size for the Public Health Question. Based on the cost-effectiveness considerations, we have adopted a cut-off value for the equivalence trial design of .06. A pairwise comparison of the $L_{mother}L_{infant}$ regimen and the $S_{mother}S_{infant}$ regimen will be used. Thus, we want to recruit enough patients to these two extreme arms of the trial to achieve a Type I error of .05 and a Type II error of .10 (power = .90) or at worst .20 (power = .80). The table below (using the formula in Blackwelder [132,133]) shows the number of patients required per treatment group for an equivalence trial with α = 5% (unilateral test), β = 10% or 20%, the critical value of δ = 6%, and different values of the common transmission rate.

Sample size per treatment group in a two-arm study

Common transmission rate	β = .10	β = .20
.08	350	253
.09	390	282
.10	428	310
.11	466	337
.12	503	363
.14	573	414
.16	640	462

With 466 evaluable patients per group randomized between the $L_{mother}L_{infant}$ arm and the $S_{mother}S_{infant}$ arm (932 total), there is a 90% power to test for equivalence when the common transmission rate is around .11 (higher than that predicted from 076). Higher power will be achieved if the common transmission rate is less than .11. Even if the common transmission rate is .16 in this study, this sample size will still provide an 80% power to test for equivalence using the 6% critical value. Thus, a total of 932 evaluable patients will provide appropriate power to detect a difference in transmission probability from .13 to .19. Smaller differences than this are not considered to be worthwhile to detect from a public health perspective. Therefore,

randomization of 932 evaluable mother-infant pairs between the Long-long and Short-short treatment groups will be sufficient to meet the study objectives for the primary hypothesis.

The 2 x 2 Factorial for the Scientific Questions. If we stopped here and conducted only a two arm trial with 932 evaluable patients randomized between $L_{mother}L_{infant}$ and $S_{mother}S_{infant}$, the trial would not provide any information about the relative importance of maternal or infant treatment duration on the observed outcome. We therefore propose a 2 x 2 factorial design which will provide information about the separate components of the treatment program using all randomized patients. Because all patients can be used for the 2 x 2 factorial analyses, the statistical power to detect treatment effects is greater than that for pairwise comparisons. By unbalancing the randomization to include two-thirds of the patients in the $L_{mother}L_{infant}$ and $S_{mother}S_{infant}$ arms, and one-third in the $L_{mother}S_{infant}$ and $S_{mother}L_{infant}$ arms we preserve the power for the primary public health question while providing an opportunity to answer the two scientific questions concerning relative treatment effect. Using this randomization scheme, the total patient enrollment will be 1400 evaluable mother-infant pairs with 466 enrolled in each of the $L_{mother}L_{infant}$ and $S_{mother}S_{infant}$ arms, and 233 enrolled in each of the $L_{mother}S_{infant}$ and $S_{mother}L_{infant}$ arms (see Study Design Figure). The randomization will be computer generated and there is no technical nor logistic complication associated with unbalancing the randomization in this study. To account for up to a 10% unevaluable rate, a total of 1556 patients will be randomized to the study.

As described by Altman [144], unequal randomization of patients between treatment arms results in a loss of power compared with equal randomization. For a two to one randomization as proposed for this study, the unequal allocation of N patients gives the same power as the equal allocation of N' = N x (8/9) patients. Thus, the statistical power for 1400 patients allocated unequally is the same as the statistical power for 1244 patients allocated equally. If there is no interaction between the effects of the maternal and infant treatment durations, the power to detect treatment effects in the 2 x 2 factorial analysis is equal to the power of a two-arm pairwise comparison with 622 evaluable patients in each arm. The sample size is sufficient to achieve 96% power to test for equivalence of treatment effects using the critical value of .06 when the common transmission probabilities are around .11. For common transmission probabilities around .16, the power is still quit high at 89%.

4.8.2 Interim Monitoring*:* This study will be monitored regularly for toxicity, compliance, and feasibility. Interim efficacy analyses will utilize formal adjustments using O'Brien-Fleming boundaries, as modified by Lan and Demets [146].

Two interim efficacy analyses and one final analysis are planned. These analyses will not be used to stop the trial early based on apparent equivalence due to the need to have firm conclusions with respect to the public health question. Additional power will be of value to convince public health officials and medical investigators about the equivalence of treatments. Furthermore, patients in the trial are not harmed by continued participation if the shorter duration therapies are as effective as the longer duration treatments.

Monitoring of transmission probabilities will therefore be conducted for patient safety. The trial will be discontinued if it is clear that any of the shorter duration treatments are inferior to the longer duration treatment. The stopping boundary will be based on the 95% upper confidence interval for each pairwise difference comparing the $L_{mother}L_{infant}$ group to each shorter duration group ($L_{mother}L_{infant}$ minus the shorter regimen). For the stopping rules, O'Brien-Fleming boundaries [145] will be used to adjust for the multiple looks, and Bonferroni adjustment will be used to adjust for the 3 pairwise comparisons with the standard. These adjustments will reduce the risk of stopping the study due to an apparent decrease in efficacy when, in fact, the efficacy is equivalent.

Two interim and one final analysis will be performed. These will be conducted when approximately 40%, 70% and 100% of the patients are evaluable. The Z-statistic boundaries for stopping the study due to clear evidence of increased transmission probability are 3.5292, 2.5795 and 2.1121, respectively. Under this monitoring scheme, if the true transmission rates are .08 for $L_{mother}L_{infant}$and .25 for $S_{mother}S_{infant}$ (the effect size estimated from ACTG 076), the chance that the study will be stopped at the first interim analysis is over 80%, and the chance that it will be stopped by the second interim analysis is over 99%. The probabilities of stopping the study early if the true transmission rates are .08 for $L_{mother}L_{infant}$ and .20 for $S_{mother}S_{infant}$ are over 40% for the first interim analysis and over 97% by the second interim analysis.

4.8.3 Analysis Plan

In general, the types of analyses planned for this study are those that were used in the analysis of protocol ACTG 076. The primary analysis of the study hypotheses will be based on HIV infection status at month 6, determined by earlier PCR analysis. Kaplan-Meier estimates of infection probabilities will be used during the conduct of this trial. When there is at least 6 months of follow-up for all infants, the analysis will be reduced to the use of simple proportions. Additional analyses will adjust for any substantial differences in baseline characteristics between the treatment groups.

We will calculate estimates of the transmission probability for each of the four arms (n=466 for the primary arms and n=233 for the secondary arms). For transmission probabilities around .11, these estimates will have standard errors of ± .0145 for the primary arms and ± .02 for the secondary arms. Thus, the study will provide precise estimates of transmission probabilities in this setting.

As suggested by Makuch and Simon [131], treatment comparisons will be based on confidence intervals for differences between the transmission probabilities. Specifically, for the public health question we will estimate the difference in transmission probabilities between the $L_{mother}L_{infant}$ and $S_{mother}S_{infant}$ treatment programs by the observed difference in transmission rate P_{Ll} - P_{Ss} based on the 932 evaluable patients. The lower 95% one-sided confidence boundary for the true difference in transmission probability between these two regimens will be calculated. If this lower confidence boundary is greater than -.06 this will be taken as evidence that reduced duration treatment is equivalent to the long duration treatment from a public health perspective. In this case we would be 95% confident that the transmission probability for the $S_{mother}S_{infant}$ treatment is not more than .06 greater than the transmission probability for the $L_{mother}L_{infant}$ treatment. The sample size for the trial provides a 90% probability that the 95% lower confidence boundary will exceed -.06 if the two transmission probabilities are equal to .11.

Treatment arms will be compared to check for appropriate balance with respect to baseline characteristics that could influence transmission probabilities. Logistic regression analyses will be used to adjust statistical inferences for these factors. The main covariates that will be considered include STDs, clinical stage of disease, CD4 count, duration of ruptured membranes, mode of delivery, duration of labor, newborn skin integrity, cigarette smoking, illicit drug use (which we assume will be extremely rare), chorioamnionitis and gestational age.

Confidence boundaries will also be calculated to answer each of the two scientific questions. These will be based on all randomized patients looking first at the influence of maternal treatment duration stratified by infant treatment duration and then at the influence of infant treatment duration stratified by maternal treatment duration. For example, the observed difference P_{Ll} - P_{Sl} and the observed difference P_{Ls} - P_{Ss} both estimate the effect on transmission probability of shortening the duration of maternal treatment. Lower 95% confidence boundaries will be calculated for each separately and a test for interaction between these two estimates will be performed. If the interaction is not significant, the estimate based on the 2 x 2 factorial design will be used to evaluate the role of maternal treatment duration. A similar analysis will be performed to evaluate the role of infant treatment duration.

Each of the final statistical analyses will be conducted at the nominal .05 level of significance without adjustment for multiple comparisons. The statistics used to answer each of the three research questions are correlated and an appropriate adjustment which is not too conservative in this setting is not clear. Furthermore, the need to adjust for multiple comparisons in final analyses is a matter of current controversy among statisticians. By focusing the study on three related questions and by identifying the primary public health question of interest, we have reduced the risk that multiple comparisons will play an important role in the final analyses. As always, caution will be needed when interpreting multiple p-values and confidence intervals.

PCR results from the peripheral blood sample drawn from the infant within 48 hours after birth will be used to estimate the proportion of transmission that occurred in utero compared with peripartum. As a working definition, we will adopt the criteria defined by Bryson et al. and endorsed at the 1993 Ghent meeting. Infants will be defined as infected in utero if the first positive PCR is found in the peripheral blood sample drawn within the first 48 hours of life. All infants who have a negative PCR at birth but who later become positive will be defined as infected at around the time of birth. The absence of breastfeeding among women in the target population for this study enables these working definitions to be applied without confounding due to post-partum transmission. The treatment arms will be compared with respect to the incidence of in utero transmission compared with peripartum transmission using logistic regression analyses. As with the analyses comparing risks of overall transmission probability, potential confounders such as maternal CD4+ lymphocyte count, p24 antigenemia, disease stage, and duration of ruptured membranes will be included in multivariate analyses of the timing of transmission.

Clinical laboratory data and adverse experiences will be examined in the analyses of safety data and will include plots of the data as well as summary statistics of the raw data and changes from baseline. Wilcoxon rank sum tests will be used to compare the groups for changes from baseline. The proportion of patients in each group who experience toxicity will be compared using a one-sided Fisher's exact test. The proportion of patients in each group reporting each individual type of adverse experience will be calculated.

4.9 Pilot Study

Safety and Tolerance of ZDV in Mothers and Infants. Two sites, one in Bangkok and one in Chiang Mai, will participate in a pilot study in order to closely monitor maternal, fetal and infant safety, and toxicity in a small number of mothers and infants (n

= 30) and to evaluate protocol interpretation, logistics, and feasibility. The protocol team and the DSMB will evaluate feasibility, data and safety profiles of the first 30 mother-infant pairs who will have completed the treatment period. Women may continue to enroll in the study during the evaluation period of the pilot.

4.10 Pharmacokinetic Studies

Pharmacokinetics of Oral ZDV during Pregnancy and at Delivery. Information on the pharmacokinetics of ZDV given orally to pregnant women during labor is limited. The first ten women enrolled in the study in Bangkok will also be enrolled in a Phase I pharmacokinetic study of oral ZDV. All will have normal renal and hepatic function and medically uncomplicated pregnancies. They will be asked to be present at the maternity unit at the earliest stages of labor. At entry they will be given the 300 mg oral loading dose of ZDV, followed by 300 mg administered in the maternity unit every 3 hours until delivery. Serial blood samples (pre dose, hours 0.5, 1, 2, 3) will be collected after every oral take to determine peak ZDV concentration and the mean terminal half-life. Maternal blood, cord blood and infant blood samples will also be obtained at the time of delivery for comparison of ZDV levels.

ZDV assays will be performed on the plasma. ZDV and Glucuronyl ZDV concentrations will also be obtained from the urine samples. Urinary excretion will be measured (total amount in urine from 0 to 3 hours).The time of the first and subsequent doses received will be accurately recorded. ZDV will be measured by Radio Immuno Assay (IRA INCSTAR Kit) using the method developed by the manufacturer.

Means and standard deviations will be calculated for peak concentration (Cmax), time to peak (Tmax), 1/2 life, area under the curve (AUC), oral and renal clearances. The t-test will be used to calculate confidence intervals for these parameters. To estimate serum half life, a linear regression of the log of the serum concentration versus time will be performed. For validation, model-based methods will be used to estimate pharmacokinetic parameters.

Population Pharmacokinetics. A two-part population pharmacokinetic study of ZDV in pregnancy and the newborn is planned. The purposes of the study are 1) to expand on the existing data on ZDV use in pregnancy and the newborn [125] and to obtain information specifically in the Thai population, 2) to supplement information on compliance, and 3) to correlate drug levels with efficacy (pharmacodynamics). In the first part of the study, the first 100 women enrolled will have timed oral dosing followed by limited serial sampling. In the second part, randomly timed samples (in relation to ingested drug) will be obtained from all women at all visits where blood samples are taken for other purposes and from all babies at the 1-month visit.

Part One. A pharmacokinetic study will be performed on the first 100 women at their 36-week visit. These mothers will be asked not to take the study drug before coming to their scheduled study visit. They will take the study drug upon arrival at the clinic and the exact time will be recorded. Within the following three hours, two blood samples (if for some reason this is not possible, at least one) will be drawn about one hour apart and the exact time will be recorded. Model-fitting will be performed using the NONMEM software package. Nonlinear mixed effect modeling (NONMEM) uses sparse data obtained randomly from a population of patients to fit kinetic models, and allows for analysis of variables that may modify the parameters. Estimates of the area under the concentration-time curve (AUC) for ZDV will be internally validated by comparison to formally calculated AUCs. In addition, the relationship between patient characteristics (dose, plasma concentrations, time course of drug exposure), virus load and infant outcome will be explored.

Part Two. As described in section 4.2.5 Post-Randomization Follow-up on Mothers and Children, women enrolled in the study will be followed monthly until the 34th week of pregnancy and then once every 2 weeks until delivery. At each visit, compliance with treatment will be assessed by pill count and a blood sample will be drawn to check toxicity. Newborns will have a blood sample taken for HIV diagnosis and for assessment of eligibility shortly after birth. After discharge from the hospital, children will continue to be followed on a monthly basis and another blood sample will be drawn at 1 month for HIV diagnosis and toxicity check-up. Serum samples will be collected at any time during the visits. At the time the sample is drawn, the exact time it is drawn will be recorded (to the closest minute), as well as the approximate time (to the closest minute) the patient took their previous two doses of study medication. AZT serum levels will be randomly assayed to evaluate compliance to the treatment. In addition, population pharmacokinetic/ pharmacodynamic modeling will be performed. Although measured drug concentration reflects only the amount of drug ingested within 3 half-lives, an overall estimate of compliance with ZDV can be obtained. This will be correlated with other covariates of drug exposure such as red cell MCV.

Although the pharmacology of ZDV in pregnant women and infants is relatively well known [125], inter-individual variation may be important to measure since ZDV is to be used in a large population. Recently published data on the short and long term effects of ZDV on virus load/replication further increase the value of population pharmacokinetic data. These data would not only provide more information on safety but also how drug exposure relates to virus load at delivery and infant outcome. Results from Part One above will be presented to the Data Safety Monitoring Board during the first interim analysis in order to assure

the safety of ZDV in women in the larger population. Routine blood samples from the remainder of the patients will be stored frozen for further compliance and pharmacokinetic/pharmacodynamic analysis.

Interpretation of Population Pharmacokinetics. The two major sources of variation in response to therapy are inter-individual variation in pharmacokinetics and variable compliance with the prescribed dosing regimen. In the case of an active control trial such as this one, assessment of compliance, and of drug exposure is very important. When the timing of the last dose is known, drug concentration from one sample can be interpreted accurately and drug exposure in individual patients can be estimated using population kinetics techniques developed by Sheiner and others [128-130]. With this technique, drug concentrations obtained at random but known intervals after dosing can be used to model population pharmacokinetic parameters. It allows the inclusion of drug measurements made at different times both within and across individuals and also permits varying numbers of sample points per individual.

A primary analysis of this data is planned as soon as the first 100 eligible subjects are accrued. The interim analysis will provide a report on the conduct of the trial and assessment of any imbalances in toxicity or kinetic data. Factors possibly responsible for altered drug clearance in subjects having an estimated AUC > 2 standard deviations from the mean of the study population will be determined. This review will be performed by the Data and Safety Monitoring Board (DSMB).

4.10 Virology Substudy

Although we believe it is quite unlikely that the short course of ZDV would be more effective than the long course, this possibility cannot be discounted. In view of recent results by Ho and Loveday [135,136], it appears that drug-resistant variants may often arise sooner than previously thought. If ZDV-resistant variants arise within three months (i.e. at peak transmission at the time of birth), viremia could conceivably be higher in women who received three months as opposed to one month of treatment [125].

To investigate the influence of virus load, biologic phenotype and genotypic ZDV resistance on the maternal to fetal transmission of HIV in the study population, a virologic substudy will be undertaken on the subset of patients enrolled at Chiang Mai University. Given the number of projected enrollees in Chiang Mai, the known seroprevalence rate in the antenatal clinic population (8%), and a projected minimal transmission rate of 8%, it is estimated that approximately 40 infants will become infected at this site. This number will be sufficient to examine the relationship of maternal and virologic characteristics with transmission. In order to execute this in a cost-effective fashion, a case control study will be established as follows: All consenting mothers enrolled at Chiang Mai will have blood drawn in "CPT" tubes and plasma and peripheral blood mononuclear leukocytes (PBLs) will be stored frozen in the laboratory in the Department of Microbiology and Immunology (See Appendix 3.1 to 3.3). These specimens will be obtained at baseline, at each follow-up visit during the antenatal period and at the time of delivery. Each mother who gives birth to an infected infant will be matched to two controls. These controls will be matched to the cases by baseline CD4 count (± 10%), age (± 5 years) and treatment assignment. Specimens from these patients will be used for the following analyses:

Relationship of Viral Load and Maternal to Fetal HIV Transmission. Higher circulating maternal viral loads have been reported to be associated with a greater risk of infant transmission of both hepatitis C virus and HIV [60,115]. In this study, maternal viral load will be assessed by the quantitative plasma HIV RNA assay developed by Roche Molecular Systems as in the ACTG (See Appendix 3.9, 3.11) [116]. This is a reverse transcription (RT) PCR-based assay that has a sensitivity of 200 RNA copies per ml of plasma. Decreases in plasma RNA are seen with initiation of antiretroviral therapy and have been associated with clinical benefit in ACTG 116B/117 [117], a trial of didanosine vs. ZDV in patients with $\geq$ 16 weeks of prior ZDV exposure and $\leq$ 300 CD4 cells/mm3. Baseline, antenatal, and time of delivery plasma specimens will be assayed in a batched fashion for each maternal case and controlled for quantitative plasma RNA. Potential correlations of baseline RNA concentration, changes after initiation of ZDV therapy and RNA concentration at the time of delivery with risk of infant transmission will be explored. Attempts to define a "threshold effect", as has been reported for HCV and suggested for HIV, will be made [60]. If such a threshold can be determined, this could serve as an important guide to antiretroviral agent management during the antepartum period.

Relationship of Biologic Phenotype and Maternal to Fetal HIV Transmission. Preliminary data have suggested that non-syncytium inducing (NSI) isolates are preferentially transmitted from mother to infant even when the mother's strain is predominantly syncytium-inducing (SI) [63,64]. To assess this in a population that is predicted to be predominantly infected with the unique E subtype of HIV-1, virus will be isolated by standard coculture techniques. Frozen PBLs from transmitting mothers and from non-transmitting mother controls, obtained at the closest time point to delivery, will be thawed and cultured by the ACTG consensus macroculture method to obtain an isolate (See Appendix 3.10)[118]. Isolates will be similarly obtained from frozen PBLs derived from infected infants. Biologic phenotyping will be done by incubating positive culture supernatants with MT-2 cells in a recently described microtiter assay [119]. Isolates will be scored as SI or NSI based on their ability to form

syncytia in MT-2 cells. The following relationships will be assessed: (1) correlation of maternal CD4 count with the presence of NSI or SI virus; (2) comparison of transmitting mothers and their non-transmitting controls for the presence of SI or NSI isolates; (3) the relationship of maternal virus load (i.e. plasma RNA concentration) with the presence of SI or NSI virus; and (4) the relationship of the biologic phenotype in transmitting mothers with their infected infants to determine if there is a predominant transmission of NSI virus.

Development of Genotypic ZDV Resistance. The ZDV-associated resistance mutation at codon 215 (Thr—>Phe or Tyr) is the key mutation among the five most commonly described mutations. It has been shown to be associated with clinical and immunologic decline and higher virus loads in HIV-infected adults [117,120,121]. Further, it has proved valuable as a marker of vertical transmission of ZDV resistant virus. Although the overall rate of ZDV resistance in the study population will predictably be low, individuals who receive the drug for up to 26 weeks may exhibit a rate of diminished ZDV susceptibility in the range of 5 to 30% depending on their stage of disease [122]. These data, derived from a North American population, provide a strong rationale for planning a targeted, cost effective investigation of the appearance of a marker of altered ZDV susceptibility in this study in Thailand. In addition to the mutation at codon 215, the mutation at codon 70 (Lys—>Arg) is of interest because it is generally the first mutation to appear and may occur within weeks of initiation of ZDV. It thus may serve as a relevant genotypic marker given the short-term ZDV exposures that women in this study will have. The nested PCR procedure of Boucher et al. (See Appendix 3.8)[123] will be employed to detect the 70 and 215 mutations in the plasma of patients . The plasma will be preferentially assayed because ZDV resistant mutations have been shown to appear in this compartment in advance of the PBL fraction [124]. Baseline and at-delivery specimens of transmitting mothers and non-transmitting controls along with neonatal samples from infected infants will be assayed for the 70 and 215 mutations. The following will be assessed: (1) the rates of development of the 70 and 215 mutations in transmitting mothers vs. non-transmitting controls; (2) the maternal virus load in mothers possessing the 70 and 215 mutations vs. those possessing purely wild type virus; and (3) the rate of vertical transmission of genotypic resistant virus and the potential discordance of fetal and maternal isolates. Since the development of ZDV resistance is a function of both stage of disease and duration of drug exposure [122], it will also be of interest to determine whether there is a difference between the two maternal treatment arms in the rate of development of genotypic resistance. For this analysis, samples from 75 mothers in each of the two maternal arms will be assayed for the 70 and 215 mutations and the rates compared. We will first look for mutations in the strata of women who have received 3 months of therapy and whose CD4 count is less than 400. If a substantial incidence of 70 and 215 mutations is found, additional studies will be considered including a search for the presence of other mutations in RT (e.g. at codon 41) and assays of phenotypic ZDV resistance. The frozen, stored material that will be available will provide a valuable specimen resource and will permit an ordered decision making process concerning the advisability of additional virologic studies. If the abbreviated ZDV regimen proves equally efficacious as the 076-like regimen in the prevention of vertical HIV transmission, the demonstration of a lower rate of development of genotypic ZDV resistance in the shorter regimen (which is likely) would be an added benefit.

4.12 Data and Safety Monitoring Board

The Data and Safety Monitoring Board (DSMB) has been specifically asked to evaluate safety and study parameters such as enrollment, compliance, follow-up, laboratory evaluations, data submission, and quality control every six months throughout the course of the study (collaboration letters included). The DSMB will decide at each of these reviews whether the study will continue as originally designed. Efficacy analyses will be conducted for two interim and one final evaluations. The interim analyses will be presented to the DSMB when 40% and 70% of the infants have been evaluated with PCR at 6 months of life. Interim analyses will utilize formal adjustments based on O'Brien-Fleming boundaries, using a Lan and DeMets Use Function.

The make up of the DSMB will be as follows: T. Brennan (Professor of Law and Public Health), S. Lagakos (Professor of Statistics), N. Bhamarapravati (Professor of Medicine), Charas Phimpilai (Medical Director of Chiang Mai McCormick Hospital). They will work independently from the research team, none of them are co-investigators in the study.

Management of the Study. The study team will consist of physicians, nurses, and social workers, in addition to the investigators. Training of all research team members will take place in the initial period of the study, and will include sessions on the theory of the subject matter of the research, practice of the procedures designed for study implementation (follow-up at home, administering questionnaires, etc.). During the first 3 to 6 months of the study, a Manual of Operations specifying procedures on all components of implementation and data handling will be reviewed. Re-training sessions will be provided every 6 months in addition to ad-hoc sessions, which will stress parts of the work that the supervisors suggest need more attention.

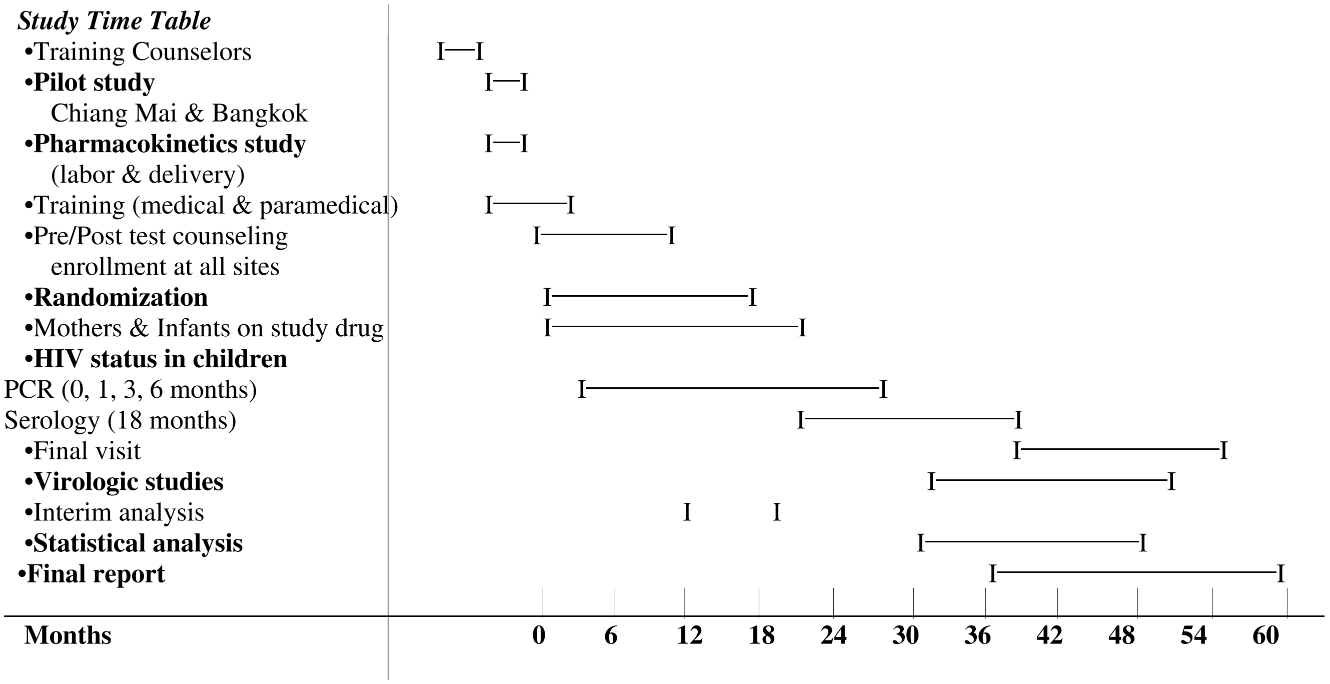

Supplement: Trial Protocol — (336 KB PDF) [file pctr.0020011.sd002.pdf]
