# Supplementary material for: Haematological Safety of Perinatal Zidovudine in Pregnant HIV-1–Infected Women in Thailand: Secondary Analysis of a Randomized Trial
Source: PLoS Clin Trials. 2007 Apr 27;2(4):e11. doi: 10.1371/journal.pctr.0020011 (PMC1863515; doi:10.1371/journal.pctr.0020011)
Supplement: Text S1 — (22 KB PDF) [file pctr.0020011.sd003.pdf]

PHPT-1 TRIAL

SECOND PRIMARY OBJECTIVES

(SAFETY AND TOLERANCE OF LONG VS. SHORTENED ZIDOVUDINE REGIMENS)

ANALYSIS PLAN

Updated, January 2007

Nelly Briand

Background

- **PHPT-1 clinical trial objectives and design**

PHPT-1 clinical trial is a phase II/III double-blind, randomized, controlled equivalence trial comparing the long standard zidovudine regimen with a shortened zidovudine regimen for the prevention of perinatal HIV-1 transmission [1]. The study was designed to test the equivalence of a short zidovudine regimen as compared to a long PACTG-076-like zidovudine regimen in reducing the risk of mother-to-child transmission of HIV.

The study had a factorial design with four arms:

- zidovudine from 28 weeks of gestation through delivery in the mothers; six weeks of zidovudine treatment for their infants;
- placebo starting at 28 weeks of gestation and then zidovudine from 35 weeks of gestation through delivery in the mothers; zidovudine for the first 3 days of life, followed by placebo through 6 weeks for their infants;
- zidovudine from 28 weeks of gestation through delivery in the mothers; zidovudine for the first 3 days of life, followed by placebo through 6 weeks for their infants;
- placebo starting at 28 weeks of gestation and then zidovudine from 35 weeks of gestation through delivery in the mothers; six weeks of zidovudine treatment for their infants.

Women were randomly assigned to one of these four groups. All infants were breastfed.

- **Zidovudine safety**

In 1994, the PACTG 076-ANRS 024 trial showed the dramatic efficacy of zidovudine for the prevention of mother-to-child transmission of human immunodeficiency virus (HIV), the rate of HIV transmission was reduced from 22.6 to 7.6 % [2;3]. Since 2004, the World Health Organization recommends zidovudine initiation at 28 weeks of gestation or as soon as possible thereafter in women who do not need treatment for their own health or when antiretroviral combinations are not available [4].

Perinatal zidovudine exposure is associated with transient anaemia and neutropenia [3;5]. Concerns about potential serious adverse effects through zidovudine

mitochondrial toxicity have been raised [6-9]. The impact of zidovudine exposure during pregnancy on haematological parameters of HIV-infected women has been assessed in several placebo-controlled studies [2;10;11]. However, these studies involved modest sample sizes, and haematological parameters were compared only at delivery.

Safety objectives

The objective of the present study is to respond to the primary safety objective of the Perinatal HIV Prevention Trial –1 (PHPT-1): to compare the safety and tolerance of long versus shortened zidovudine regimens. Safety and tolerance of each regimen will be evaluated by examining adverse events, laboratory and clinical data of the mothers, particularly their haematological parameters such as haemoglobin level, leucocytes, neutrophils and lymphocytes.

In the present study we will assess the evolution of haematological parameters in HIV-infected women, including haemoglobin level, leucocytes, absolute neutrophil counts and total lymphocyte counts, from 26 weeks of gestation to delivery.

Statistical methods

- **PHPT-1 dataset**

The data set includes data keyed from all cases report forms completed by the site investigators. After double data entry, data sets were checked for unusual values, inconsistencies between fields. Queries to the co-investigators were generated as necessary. As required by Good Clinical Practices, any change made to the original data set was fully documented.

- **Parameters for the analysis**

Maternal blood samples collected at:

- Pre-entry visit, planned at 26 weeks of gestation,
- 32 weeks of gestation,
- 35 weeks of gestation,
- delivery (upon admission to the maternity unit).

Haematological measurements considered:

- haemoglobin level,
- leucocytes,
- absolute neutrophil counts,
- total lymphocyte counts.

Analysed as:

- quantitative variables: means and medians,
- qualitative variables: percentages of women with various grades of toxicity severity (NIH, NIAID, Division of AIDS tables)

- **Management of missing or inconsistent data**

Haematological values for each woman will be plotted in a chart. Any value above or below 3 standard deviations of the mean will be checked against the original laboratory result slip.

- **Statistical methods and tests**

Comparison according to the randomization arms:

- Observed haematological parameters at each visit (baseline, 32 weeks, 35 weeks and delivery) using Student's T-test (quantitative variables) or Fisher's test (qualitative variables),
- Changes in haematological parameters from 26 weeks of gestation to delivery, using mixed linear models to take into account repeated measures.

Covariates collected at baseline:

- Socio-demographic: maternal age, body mass index, parity, education level, region of residence;
- Biological and clinical characteristics: HIV clinical stage, CD4 count and HIV-1 RNA viral load.

The analysis will follow the principle of intention-to-treat.

Dropouts will be taken into account with the complete data method.

Women who are transfused for anaemia or haemorrhage during pregnancy will be treated on a case-by-case basis.

No correction for multiple comparisons will be performed.

Any ad-hoc subgroup analyses will be thoroughly discussed if needed.

The p-values reported will be two-tailed and an alpha level of 0.05 will be used to assess statistical significance.

The goodness of fit of the models will be checked by plotting the residuals.

References

1. Lallemant M, Jourdain G, Le Coeur S *et al*. A trial of shortened zidovudine regimens to prevent mother-to-child transmission of human immunodeficiency virus type 1. Perinatal HIV Prevention Trial (Thailand) Investigators. N Engl J Med. 2000; 343(14):982-91.
2. Connor EM, Sperling RS, Gelber R *et al*. Reduction of maternal-infant transmission of human immunodeficiency virus type 1 with zidovudine treatment. Pediatric AIDS Clinical Trials Group Protocol 076 Study Group. N Engl J Med. 1994; 331(18):1173-80.
3. Sperling RS, Shapiro DE, McSherry GD *et al*. Safety of the maternal-infant zidovudine regimen utilized in the Pediatric AIDS Clinical Trial Group 076 Study. AIDS. 1998; 12(14):1805-13.
4. Scaling up antiretroviral therapy in resource-limited setting: treatment guidelines for a public approach, 2003 revision. Annex E: WHO staging system for HIV infection and disease in adults and adolescents. Geneva: World Health Organization, 2004.
5. Culnane M, Fowler M, Lee SS *et al*. Lack of long-term effects of in utero exposure to zidovudine among uninfected children born to HIV-infected women. Pediatric AIDS Clinical Trials Group Protocol 219/076 Teams. JAMA. 1999; 281(2):151-7.
6. Blanche S, Tardieu M, Rustin P *et al*. Persistent mitochondrial dysfunction and perinatal exposure to antiretroviral nucleoside analogues. Lancet. 1999; 354(9184):1084-9.
7. Barret B, Tardieu M, Rustin P *et al*. Persistent mitochondrial dysfunction in HIV-1-exposed but uninfected infants: clinical screening in a large prospective cohort. AIDS. 2003; 17(12):1769-85.
8. Dalakas MC, Illa I, Pezeshkpour GH, Laukaitis JP, Cohen B, Griffin JL. Mitochondrial myopathy caused by long-term zidovudine therapy. N Engl J Med. 1990; 322(16):1098-105.
9. Poirier MC, Divi RL, Al-Harthi L *et al*. Long-term mitochondrial toxicity in HIV-uninfected infants born to HIV-infected mothers. J Acquir Immune Defic Syndr. 2003; 33(2):175-83.
10. Wiktor SZ, Ekpini E, Karon JM *et al*. Short-course oral zidovudine for prevention

of mother-to-child transmission of HIV-1 in Abidjan, Cote d'Ivoire: a randomised trial. Lancet. 1999; 353(9155):781-5.

11. Shaffer N, Chuachoowong R, Mock PA *et al*. Short-course zidovudine for perinatal HIV-1 transmission in Bangkok, Thailand: a randomised controlled trial. Bangkok Collaborative Perinatal HIV Transmission Study Group. Lancet. 1999; 353(9155):773-80.
